# Novel Drug-like HsrA Inhibitors Exhibit Potent Narrow-Spectrum Antimicrobial Activities against *Helicobacter pylori*

**DOI:** 10.3390/ijms251810175

**Published:** 2024-09-22

**Authors:** Javier Casado, Irene Olivan-Muro, Sonia Algarate, Eduardo Chueca, Sandra Salillas, Adrián Velázquez-Campoy, Elena Piazuelo, María F. Fillat, Javier Sancho, Ángel Lanas, Andrés González

**Affiliations:** 1 Group of Translational Research in Digestive Disease, Institute for Health Research Aragón (IIS Aragón), San Juan Bosco 13, 50009 Zaragoza, Spain; jcasado@unizar.es (J.C.); educhuec@gmail.com (E.C.); epiazor@unizar.es (E.P.); alanas@unizar.es (Á.L.); 2 Department of Biochemistry and Molecular & Cellular Biology, University of Zaragoza, Pedro Cerbuna 12, 50009 Zaragoza, Spain; olivanmuro@unizar.es (I.O.-M.); sandrasalillasberges@gmail.com (S.S.); adrianvc@unizar.es (A.V.-C.); fillat@unizar.es (M.F.F.); jsancho@unizar.es (J.S.); 3 Institute for Biocomputation and Physics of Complex Systems (BIFI), Mariano Esquilor (Edif. I+D), 50018 Zaragoza, Spain; 4 Microbiology Service, University Clinic Hospital Lozano Blesa, San Juan Bosco 15, 50009 Zaragoza, Spain; sonialgarate@gmail.com; 5 Biomedical Research Networking Centre in Hepatic and Digestive Diseases (CIBERehd), Monforte de Lemos 3-5, 28029 Madrid, Spain; 6 Aragón Health Sciences Institute (IACS), San Juan Bosco 13, 50009 Zaragoza, Spain; 7 Department of Medicine, Psychiatry and Dermatology, University of Zaragoza, Pedro Cerbuna 12, 50009 Zaragoza, Spain; 8 Digestive Diseases Service, University Clinic Hospital Lozano Blesa, San Juan Bosco 15, 50009 Zaragoza, Spain

**Keywords:** *Helicobacter pylori*, antimicrobial resistance, narrow-spectrum antibiotic, antibacterial target, response regulator, HsrA, *Campylobacter jejuni*

## Abstract

*Helicobacter pylori* infection constitutes a silent pandemic of global concern. In the last decades, the alarming increase in multidrug resistance evolved by this pathogen has led to a marked drop in the eradication rates of traditional therapies worldwide. By using a high-throughput screening strategy, in combination with in vitro DNA binding assays and antibacterial activity testing, we identified a battery of novel drug-like HsrA inhibitors with MIC values ranging from 0.031 to 4 mg/L against several antibiotic-resistant strains of *H. pylori*, and minor effects against both Gram-negative and Gram-positive species of human microbiota. The most potent anti-*H. pylori* candidate demonstrated a high therapeutic index, an additive effect in combination with metronidazole and clarithromycin as well as a strong antimicrobial action against *Campylobacter jejuni*, another clinically relevant pathogen of phylum *Campylobacterota*. Transcriptomic analysis suggests that the in vivo inhibition of HsrA triggers lethal global disturbances in *H. pylori* physiology including the arrest of protein biosynthesis, malfunction of respiratory chain, detriment in ATP generation, and oxidative stress. The novel drug-like HsrA inhibitors described here constitute valuable candidates to a new family of narrow-spectrum antibiotics that allow overcoming the current resistome, protecting from dysbiosis, and increasing therapeutic options for novel personalized treatments against *H. pylori*.

## 1. Introduction

*Helicobacter pylori*, a pathogenic member of the phylum *Campylobacterota* (formerly *Epsilonproteobacteria*) [1], is recognized as the leading cause of several human gastric pathologies including acute and chronic gastritis, peptic ulcer disease, gastric adenocarcinoma, and gastric mucosa-associated lymphoid tissue (MALT) lymphoma [2,3]. In addition, this microaerophilic Gram-negative bacterium has been associated with some extragastric disorders such as idiopathic thrombocytopenic purpura, idiopathic iron deficiency anemia, vitamin B_12_ deficiency as well as cardiovascular and neurodegenerative diseases, among others [4]. *H. pylori* is presumably colonizing the gastric mucosa of more than half of the world’s population [5], causing a chronic long-lasting inflammation that may progress to gastric cancer in about 1–3% of untreated or mistreated cases [6,7]. Despite the fact that *H. pylori* constitutes the only bacterial pathogen recognized as a Class I carcinogen by the International Agency for Research on Cancer [8], neither prophylactic nor therapeutic vaccines are currently available. 

Effective treatment of *H. pylori* infection remains a challenge for clinicians and sanitary authorities worldwide [9,10,11]. Only a handful of antibiotics are currently used in clinical practice for *H. pylori* eradication including metronidazole, clarithromycin, amoxicillin, tetracycline, and levofloxacin [12,13]. Due to the poor in vivo efficacy of antimicrobial monotherapies against this pathogen [14], current antibiotics are administered as part of several aggressive and prolonged combinatory therapies that are commonly prescribed empirically, frequently resulting in *H. pylori* eradication failures, the emergence of secondary antibiotic resistances, and refractory infections [15,16,17,18]. In the last two decades, the alarming increase in antibiotic resistance levels to first-line and even “rescue” antibiotics, especially clarithromycin, metronidazole, and levofloxacin [19,20,21], has led to a marked decrease in the eradication rates of traditional therapies [22,23]. This fact has prompted the World Health Organization (WHO) to define *H. pylori* as a high priority pathogen in the current global efforts in the R&D of novel antimicrobials [24]. 

On the other hand, even when current antimicrobial therapies against *H. pylori* infections are generally safe, the prolonged administration of high doses of broad-spectrum antibiotics in combinatory therapies frequently result in dysbiosis [25,26,27,28]. Thus, standard triple therapy, sequential therapy, bismuth-based quadruple therapy, and concomitant therapy [12,13] induced long-term changes in the gut microbiota of Chinese children [28]. In some cases, *H. pylori* eradication regimens might cause a major disruption of the normal gut microbiome, promoting severe complications such as pseudomembranous colitis due to *Clostridioides difficile* proliferation [29,30]. Fulminant *C. difficile* colitis after the administration of *H. pylori* eradication therapies have been documented [31,32]. 

The discovery and development of narrow-spectrum antibiotics, also known as precision antimicrobials, exemplify the current accurate strategy for reducing or slowing down the appearance and propagation of antimicrobial resistance as well as minimizing undesirable side effects on the normal microbiome [33,34,35,36,37]. Ideally, a precision antimicrobial exerts its action on molecular targets shared by only a reduced number of closely related pathogenic species, but not expressed by most representative commensal microbes [38,39]. 

In previous works, we have validated the essential protein HsrA as an effective therapeutic target for *H. pylori* infection [40,41,42]. HsrA (also known as HP1043) is an OmpR/PhoB-type orphan response regulator [43,44] that is unique and highly conserved among members of the phylum *Campylobacterota* [45]. The regulator functions as a dimeric protein in vivo preferably acting as a transcriptional activator, modulating its own expression but also the transcription of a variety of genes involved in crucial physiological processes including transcription, translation, redox homeostasis, chemotaxis, and energy metabolism [46,47,48,49], thereby resulting in being indispensable for cell viability [50,51]. The expression level of this response regulator appears relevant for *H. pylori* homeostasis; both *hsrA* deletion and protein overexpression have been unsuccessful [45,46]. Several studies suggest that HsrA acts as a homeostatic stress regulator, coordinating cell division, metabolic functions and oxidative stress defenses in response to nutrient availability and toxic metabolites [47,48,49]. Structural studies have demonstrated that HsrA contains two functional domains, an N-terminal regulatory domain and a C-terminal DNA-binding/effector domain. However, the protein functions in a phosphorylation-independent manner, and mutations in the N-terminal regulatory domain did not affect its DNA binding activity [43,45].

In the present study, we identified several novel low-molecular weight inhibitors of HsrA, which fit the Lipinski’s rule-of-five for oral drug-likeness and exhibited potent bactericidal activities against *H. pylori*, with minor antimicrobial effects on both Gram-negative and Gram-positive species of commensal bacteria. At least one of these novel precision antimicrobials also exerted a strong antimicrobial action against *Campylobacter jejuni*, another clinically relevant pathogen of phylum *Campylobacterota*.

## 2. Results

### 2.1. High-Throughput Screening of the Maybridge HitFinder^TM^ Chemical Library Identified Novel HsrA Ligands

**Figure 1:**
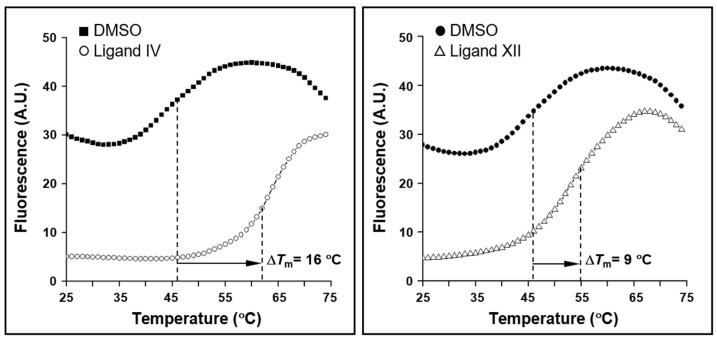
Upshifts in the thermal unfolding curve of response regulator HsrA triggered by ligands IV and XII. The high-throughput screening (HTS) of the Maybridge HitFinder^TM^ chemical library for HsrA ligands was assessed by a fluorescence-based thermal shift assay. Mixtures of DMSO (vehicle) instead of compounds were used as reference controls. Any compound that preferentially binds to the native state of HsrA would increase protein stability and cause an increase in the midpoint temperature of unfolding (*T*_m_). Compounds that clearly increased the thermal stability of HsrA observed in the reference controls (*T*_m_ ≥ 5 °C) were considered as HsrA ligands.

The Maybridge HitFinder^TM^ chemical collection, comprising 12,000 compounds that fit Lipinski’s rule-of-five for oral drug-likeness [52,53], was screened for the identification of low-molecular weight ligands of the *H. pylori* essential response regulator HsrA. The high-throughput screening (HTS) of the chemical library was carried out by using a previously described fluorescent thermal shift-based method [40,54]. With this approach, compounds from the library that induced a thermostabilization of the protein in ≥5 °C compared to reference controls with dimethyl sulfoxide (DMSO, vehicle) were considered as HsrA ligands (Figure 1). According to this selection criterion, our HTS led to the identification of 31 drug-like small molecules from the Maybridge collection than acted as ligands of HsrA (Supplementary Table S1). For practical purposes, the HsrA ligands were identified by consecutive Roman numerals according to the magnitude of the melting temperature (*T*_m_) upshifts that they induced after binding to the native state of the protein. Almost a third of the HsrA ligands identified by the HTS induced *T*_m_ upshifted above 10 °C. Notably, two HsrA-ligand complexes increased the protein thermal stability at 20 °C or more. Chemical structures and some physicochemical properties of the most relevant HsrA ligands identified and evaluated in this work are shown in Table 1.

### 2.2. Several Drug-like Ligands of HsrA Inhibited Its DNA Binding Activity In Vitro

**Figure 2:**
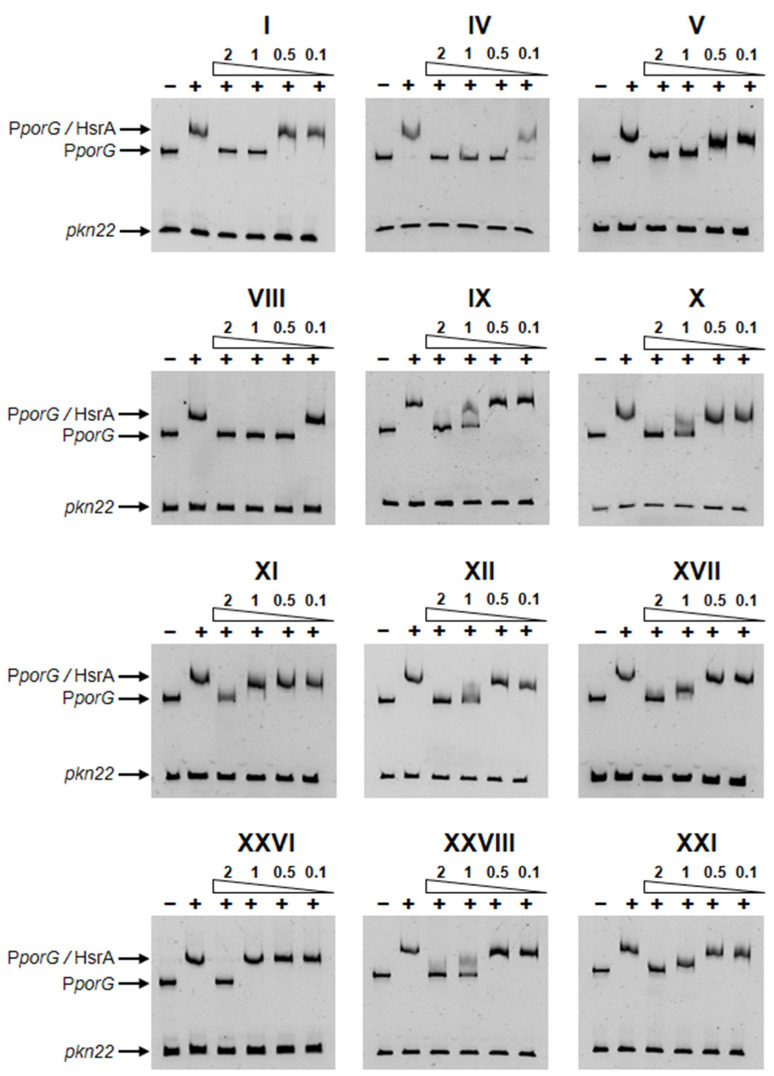
Specific in vitro inhibition of the DNA binding activity of response regulator HsrA by the selected ligands, according to the electrophoretic mobility shift assays (EMSAs). Recombinant HsrA was mixed with its target promoter P*porG* in the presence of 2, 1, 0.5, and 0.1 mM of ligands. For practical purposes, the HsrA ligands have been identified by consecutive Roman numerals according to the magnitude of the melting temperature (*T*_m_) upshifts that they induced after binding to the native state of the protein (see Supplementary Table S1). DMSO instead of inhibitors was included as the vehicle control, while an internal sequence of the *Anabaena* sp. *pkn22* gene was used as a non-specific competitor in all assays. The addition of protein in the reaction mixtures is indicated by +. Protein-DNA interactions were analyzed by 6% non-denaturing electrophoresis.

The inhibitory capabilities of all HsrA ligands identified by the HTS on the in vitro DNA binding activity of the response regulator were evaluated by electrophoretic mobility shift assays (EMSAs). Previous works have demonstrated a high affinity of HsrA in vitro for its target promoter P*_porGDAB_* in a concentration-dependent manner [40,41]. In the present work, titration of the HsrA activity confirmed that 120 ng of target DNA were completely and specifically complexed with at least 6 μM of freshly purified recombinant protein under the experimental conditions used in EMSA. Accordingly, mixtures of these amounts of protein and target DNA were incubated in the presence of increasing concentrations of each HsrA ligand from 100 μM to 2 mM. Twelve of the thirty-one low-molecular weight ligands of HsrA caused a noticeable inhibition of the in vitro DNA binding activity of this essential response regulator according to the EMSAs (Figure 2). Despite 2 mM of any of these compounds being sufficient to completely inhibit the in vitro biological activity of 6 μM of recombinant HsrA protein, the in vitro inhibitory effects of some ligands like IV and VIII appeared appreciably stronger than those exerted by other ligands such as XI and XXVI.

### 2.3. Novel HsrA Inhibitors Exhibited Potent Bactericidal Effects against H. pylori

The twelve new drug-like HsrA inhibitors identified by HTS and EMSA were evaluated for their antimicrobial properties against four different strains of *H. pylori* including strains resistant to clarithromycin (ATCC 700684), metronidazole (ATCC 43504), and levofloxacin (strain Donostia 2). As shown in Table 2, at least six HsrA inhibitors (I, IV, V, VIII, XI, and XII) exhibited strong bactericidal activities against all of the *H. pylori* strains tested, showing minimal inhibitory concentration (MIC) values ranging from 0.031 to 4 mg/L. Notably, HsrA ligand V appeared especially effective against *H. pylori* viability, exhibiting a bactericidal potency even stronger than those demonstrated by first-line antibiotics like metronidazole and levofloxacin against the same *H. pylori* strains. Other HsrA inhibitors such as IX, X, XVII, and XXVI demonstrated moderate anti-*H. pylori* action (MIC = 8–16 mg/L), while inhibitors XXVIII and XXXI appeared to be poorly effective as antimicrobials (MIC ≥ 32 mg/L). No relevant differences were observed in the antimicrobial activities of these molecules with respect to the antibiotic-resistance pattern of the *H. pylori* strains used in the assays.

**Figure 3:**
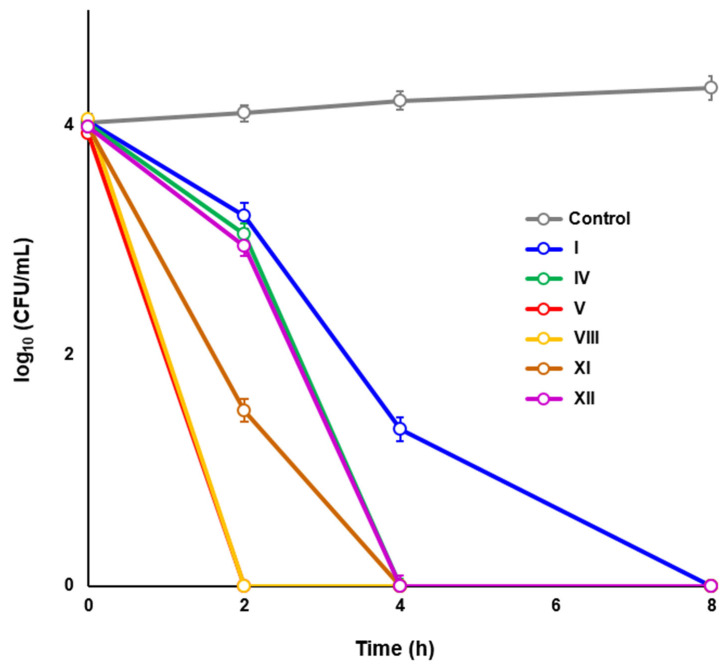
Time-kill kinetics of selected HsrA inhibitors against *Helicobacter pylori* strain ATCC 700392. Fresh grown cells of *H. pylori* (10^4^ CFU/mL) were exposed to 4-fold the value of thee minimal inhibitory concentration (MIC) of each compound. Mixtures of bacteria with DMSO (vehicle) instead of inhibitors were used as controls. CFUs were determined at time zero and after 2, 4, 8, and 24 h of exposure. Values are the means of three independent determinations; standard deviations are represented by vertical bars. Differences in *H. pylori* survival were subjected to statistical analysis by using the Mann–Whitney *U* test.

To further characterize the bactericidal activities of the most effective HsrA inhibitors, time-kill kinetic assays were carried out by exposing the *H. pylori* strain ATCC 700392 to 4 × MIC of compounds I, IV, V, VIII, XI, and XII (Figure 3). Although all compounds resulted in being completely lethal after 8 h of exposure, significant differences (*p* < 0.05) were noticed in the decline in the bacterial counts produced by each HsrA inhibitor from 2 h of treatment. Thus, at the concentration used in this experiment, compounds V and VIII completely killed 10^4^ *H. pylori* cells after 2 h of exposure. The same total bactericidal effect was observed after 4 h for compounds IV, XI, and XII. No live bacteria could be detected after 8 h of exposure to compound I. 

Putative combinatory effects in the antimicrobial action of these bactericidal HsrA inhibitors with first-line antibiotics including clarithromycin, metronidazole, and levofloxacin against *H. pylori* were assessed by checkerboard assays. As described in Table 3, only compound XII exhibited a synergistic effect in combination with metronidazole, while compound IV did not demonstrate an interaction with any of the antibiotics analyzed. The highly bactericidal compounds V, VIII, and XI interacted additively with both clarithromycin and metronidazole; however, all HsrA inhibitors resulted in being neutral in combination with levofloxacin.

### 2.4. An HsrA Bactericidal Inhibitor Bound the HsrA Ortholog Protein CosR and Exhibited Strong Bactericidal Effect against C. jejuni

Since HsrA is unique and highly conserved among members of the phylum *Campylobacterota*, HsrA inhibitors that previously demonstrated strong bactericidal activities against *H. pylori* (MIC ≤ 4 mg/L) were additionally evaluated for their antimicrobial properties against another *Campylobacterota* pathogenic species, *C. jejuni*. Notably, the most effective HsrA inhibitor against *H. pylori*, ligand V, also exhibited a strong antimicrobial activity against *C. jejuni*, with MIC and MBC values ≤ 0.5 mg/L (Table 4). However, the antimicrobial effect of other HsrA ligands against *C. jejuni* appeared to be moderate (16–32 mg/L) or quite low (≥64 mg/L) for the rest of compounds. 

**Figure 4:**
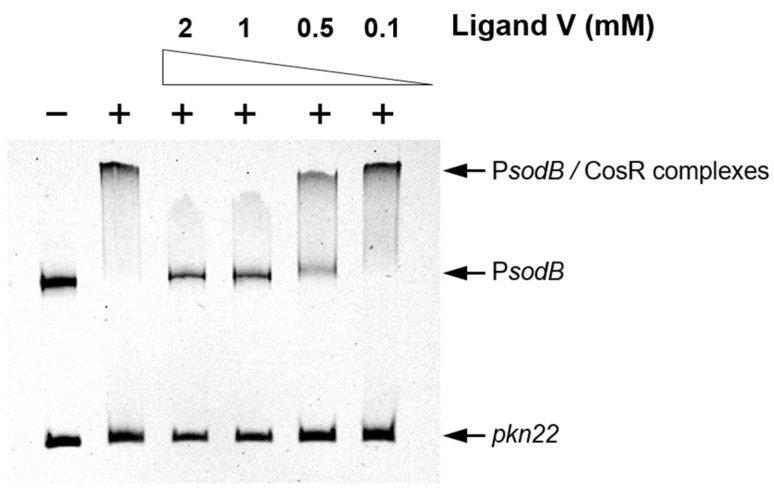
HsrA ligand V noticeably inhibited the in vitro DNA binding activity of the *C. jejuni* response regulator CosR, an ortholog protein of HsrA. Recombinant CosR was mixed with its target promoter P*sodB* in the presence of 2, 1, 0.5, and 0.1 mM of ligand V. DMSO instead of the inhibitor was included as the vehicle control, while an internal sequence of the *Anabaena* sp. *pkn22* gene was used as a non-specific competitor in all assays. The addition of protein in the reaction mixtures is indicated by +. Protein–DNA interactions were analyzed by 6% non-denaturing electrophoresis.

Considering that the essential response regulator CosR constitutes the ortholog of HsrA in *C. jejuni* [44,45], we analyzed the effect of ligand V on the in vitro DNA binding activity of CosR by EMSAs. As depicted in Figure 4, the HsrA inhibitor V also caused a noticeable inhibition in the in vitro affinity of CosR by its target promoter P*sodB*, suggesting that this low-molecular weight compound acts as an inhibitory ligand of both orthologs, HsrA and CosR.

### 2.5. HsrA Bactericidal Inhibitors Exhibited Low Antimicrobial Actions against Gram-Positive and Gram-Negative Species of Human Microbiota

Furthermore, we evaluated the antimicrobial activities of the most potent bactericidal anti-*H. pylori* inhibitors against several Gram-negative and Gram-positive representative species of the human normal microbiota. MIC and MBC values of HsrA ligands I, IV, V, VIII, XI, and XII against control strains of *E. coli*, *K. pneumoniae*, *E. faecalis*, *S. aureus*, *S. epidermidis*, and *S. agalactiae* were determined according to the EUCAST guidelines. As shown in Table 4, most of the HsrA inhibitors exhibited a narrow spectrum of action against *H. pylori*, and did not exert relevant antimicrobial effects against any of the microbiota species tested. Unexpectedly, compounds VIII and XI appeared to be particularly effective against *Staphylococcus* sp. and *Streptococcus* sp. strains, which could suggest cross-inhibition of specific molecular targets expressed by these bacterial species.

### 2.6. Cytotoxicity and Therapeutic Index

**Figure 5:**
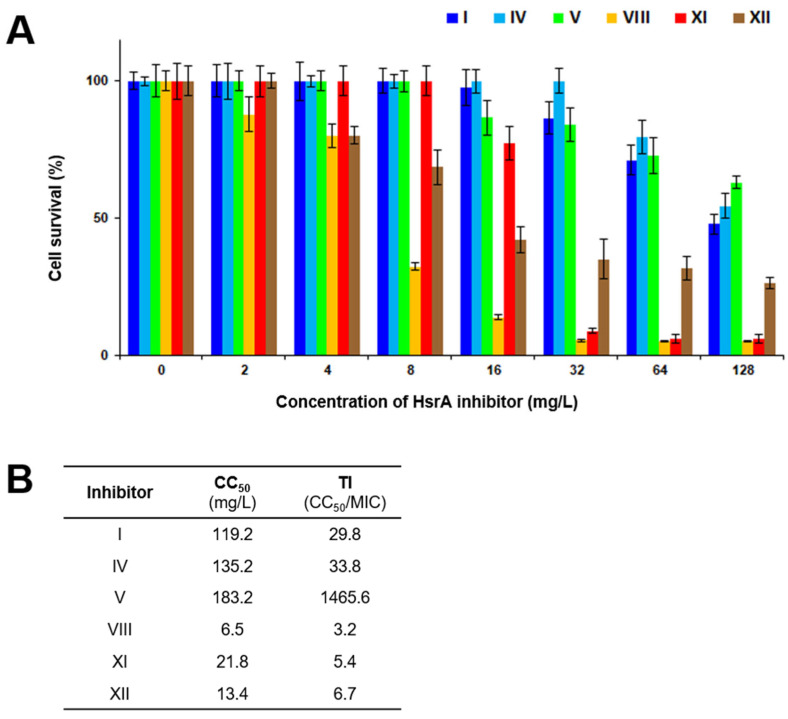
Cytotoxicity and therapeutic index of selected highly bactericidal HsrA inhibitors. (**A**) The cytotoxicity of HsrA inhibitors I, IV, V, VIII, XI, and XII toward HeLa cells was assessed at 24 h of exposure through the PrestoBlue^TM^ method. Experiments were performed twice in triplicate, vertical bars represent standard deviations. (**B**) The 50% cytotoxic concentration (CC_50_) was defined as the ligand concentration that reduced the viability of the DMSO (vehicle)-treated cell cultures by 50%. Therapeutic index (TI) of each compound was calculated as the ratio between CC_50_ and the minimal inhibitory concentration (MIC).

As a preliminary study of toxicity, the cytotoxicity effects of highly bactericidal anti-*H. pylori* inhibitors I, IV, V, VIII, XI, and XII were evaluated on HeLa cells. Mammalian cells were exposed over 24 h to increasing concentrations of each compound, from 0.125 to 128 mg/L, and cell viability was measured by the PrestoBlue^TM^ method. None of the six HsrA inhibitors resulted in being cytotoxic for Hela cells under 2 mg/L, while compounds I, IV, V, and XI seemed to be completely safe even at 8 mg/L (Figure 5A). Compound VIII proved to be the most cytotoxic among the highly bactericidal HsrA ligands, causing 68% of cell death at 8 mg/L. Notably, the 50% cytotoxic concentrations (CC_50_) of compounds I, IV, and V were 30 times or higher their values of MIC (Figure 5B), which supposes wide therapeutic windows of these novel anti-*H. pylori* antimicrobial candidates. Surprisingly, the most potent bactericidal HsrA ligand was also the safest candidate.

### 2.7. Thermodynamics and Predicted Models of HsrA-Inhibitor Interactions

Isothermal titration calorimetry (ITC) experiments and molecular docking analyses were combined in order to achieve a better understanding of the thermodynamic, stoichiometry, and putative structures of HsrA-inhibitor complexes. As shown in Table 5, all highly bactericidal inhibitors of HsrA appeared to interact with amino acid residues involved in the helix-turn-helix (HTH) DNA binding motif of the regulator and their immediate environments, following a 1:1 stoichiometry. Thus, each HsrA monomer binds one molecule of inhibitor with dissociation constants in the micromolar range. Ligands XI and XII exhibited significant lower binding affinities to HsrA according to their *K*_d_ values (Table 5, Supplementary Figure S1). ITC experiments also confirmed the molecular interaction of highly bactericidal ligand V with the ortholog of HsrA in *C. jejuni*, the orphan response regulator CosR (Supplementary Figure S2). This low-molecular weight inhibitor of HsrA bound to CosR with moderate affinity, showing a dissociation constant in the micromolar range and followed a 1:1 stoichiometry. 

**Figure 6:**
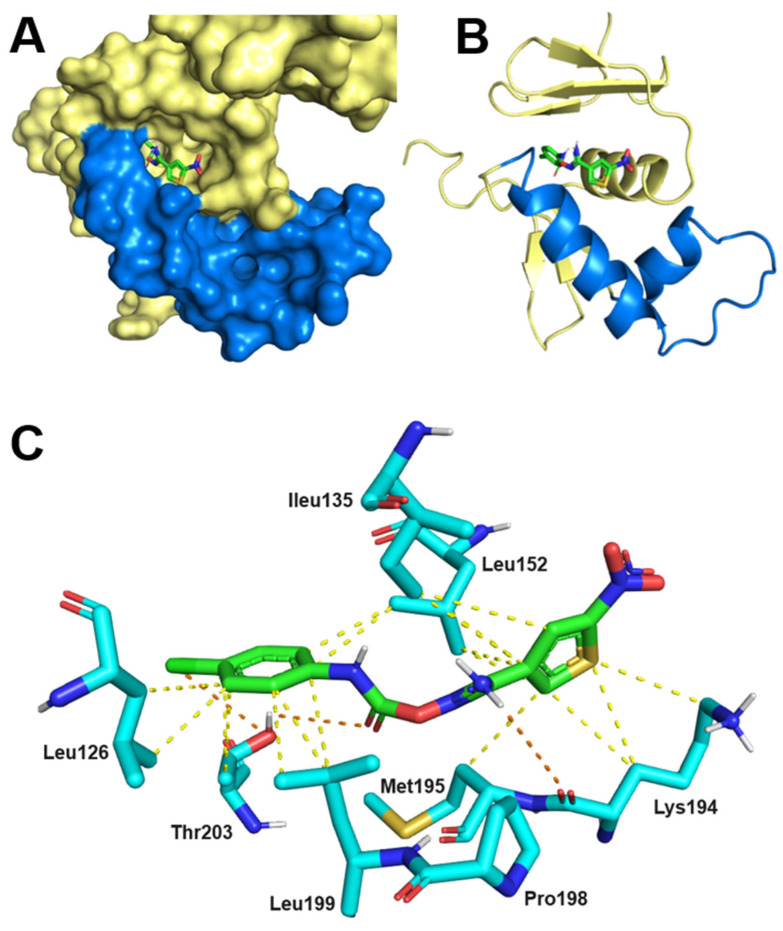
Predicted model of molecular interaction between HsrA and ligand V. (**A**) 3D view (molecular surface) and (**B**) ribbon diagram of the HsrA effector domain and the best-ranked docking pose of ligand V. The helix-turn-helix (HTH) DNA binding motif has been highlighted in blue. (**C**) Detailed view of the amino acid residues directly involved in the interaction of HsrA with ligand V. Hydrophobic interactions are indicated with yellow dashed lines, H-bonds and halogen bonds are indicated with orange dashed lines. Molecular docking analysis was performed by AutoDock Vina “vina.scripps.edu (accessed on 19 September 2024)”. Protein-ligand complex was visualized by PyMOL “www.pymol.org (accessed on 19 September 2024)”.

As previously observed with other HsrA low-molecular weight ligands [42], the novel drug-like inhibitors described here presumably interact with the C-terminal DNA binding domain of HsrA at a putative binding pocket, predominantly shaped by the nonpolar residues I121, I123, I126, I135, V142, V144, G146, P148, F149, L152, M195, P198, and L199, with a few polar amino acids such as Y137, K145, and K194. Inside this predicted binding pocket, the best-ranked pose of the most effective HsrA bactericidal inhibitor against *H. pylori*, ligand V, appears to establish several non-covalent interactions with neighboring amino acids that boost the stabilization of the ligand-protein complex (Figure 6). Thus, the phenyl ring of ligand V establishes CH/π interactions with residues Leu126, Thr203, Leu199, and Leu152 (Figure 6C). In addition, the hydroxyl group in the side chain of Thr203 interacts through hydrogen bonding with the carbonyl group of ligand V, and through halogen bonding with the chloride substituent located in the *para*-position of the ligand phenyl group. A series of hydrophobic interactions are formed between the heterocyclic thiophene group of ligand V and residues Lys194, Met195, Leu152, and Ileu135. Furthermore, the NH_2_ group of ligand V establishes a hydrogen bond with the carbonyl group of Lys194, while the aromatic ring of Pro198 could interact with thiophene through perpendicular π/π stacking at a 4.5 Å distance. In the best-ranked pose of ligand V into the pocket of the C-terminal DNA binding domain of HsrA, the nitro group at the C2 position in the thiophene of the ligand appears to be located extremely outside the binding pocket and therefore too far away from the amino groups of Leu152 and Lys194 to establish any interaction (Figure 6A,C).

### 2.8. In Vivo Inhibition of HsrA Uncovered New Insights into the Essential Role of This Orphan Response Regulator

**Figure 7:**
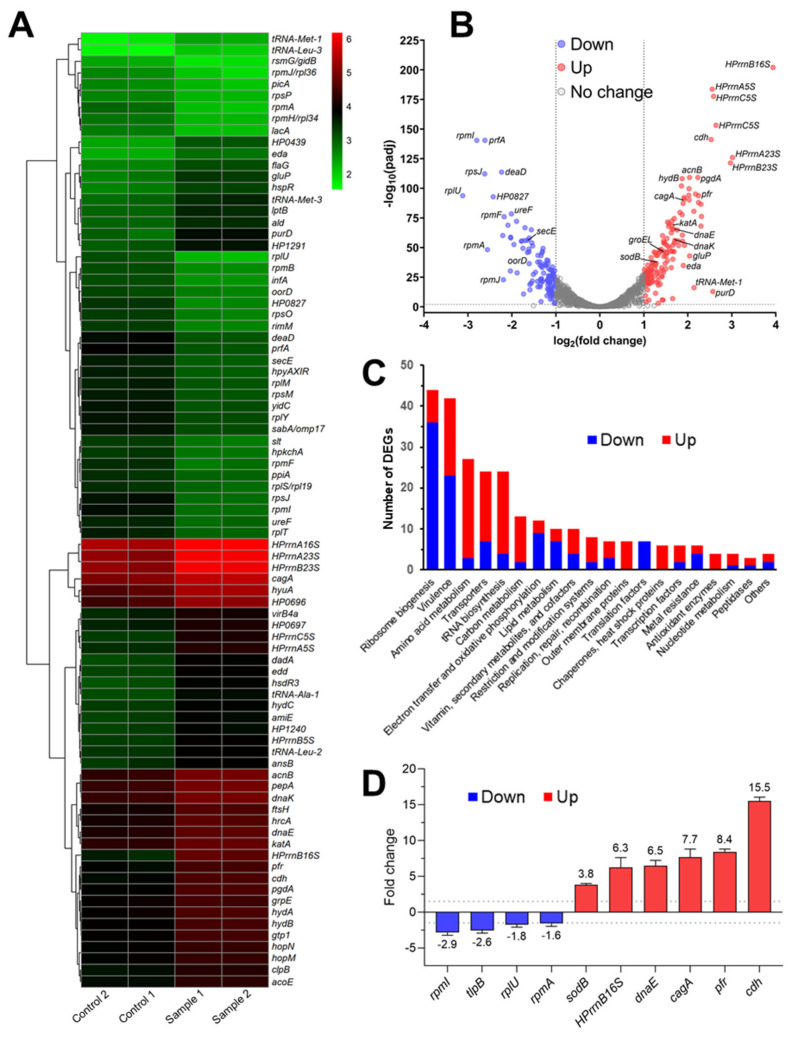
Transcriptomic analysis of *H. pylori* cells exposed to lethal concentrations of HsrA bactericidal inhibitor V. Fresh cultures of *H. pylori* 26695 adjusted to 10^7^ CFU/mL were exposed in triplicate to 4-fold the value of the minimal inhibitory concentration of HsrA inhibitor V and incubated under controlled conditions (microaerobiosis, 37 °C) until the log_10_ CFU/mL resulted in being diminished in one unit. (**A**) Heat map showing the hierarchical cluster analysis of selected differentially expressed genes (DEG, absolute log2 fold change >1 and *p*-value < 0.05). Each row represents a gene, and each column represents a sample. Red and green indicate upregulated and downregulated genes, respectively. The gradient color barcode at the top right indicates the log_10_ normalized hit counts. (**B**) Volcano plot indicating the distribution of DEGs. (**C**) Distribution of upregulated (column in red) and downregulated (column in blue) known-function genes by functional categories. (**D**) qPCR analysis of selected genes. Dashed grey lines indicate fold change cut-off values of ±1.5.

The inhibitory effect of the highly bactericidal inhibitor V on the essential transcriptional regulatory role of HsrA in vivo was assessed by RNA-Seq. For this purpose, ~3 × 10^7^ CFU/mL freshly grown *H. pylori* 26695 cells were exposed to lethal concentrations of compound V (4 × MIC) until the log_10_ CFU/mL resulted in being diminished in one unit (~2 h of exposure). As depicted in Figure 7, the transcriptomic analysis suggested that the in vivo inhibition of HsrA induced a global misregulation of *H. pylori* gene expression, which appears to be conducive to cell death as a result of several serious and possibly synergistic physiological alterations. 

Treatment with the bactericidal HsrA inhibitor V significantly changed the transcript levels of 367 ORF (absolute log2 fold change > 1, *p*-value < 0.05), of which 212 genes appeared to be upregulated and 155 genes resulted in downregulation compared with the control samples (Figure 7A,B, Supplementary Table S2). Thus, in vivo HsrA inhibition seems to influence, directly or indirectly, the expression of 23% of ORFs encoded by the *H. pylori* 26696 genome [55,56]. Among the 268 differentially expressed genes (DEGs) with defined functions, two functional categories were highly enriched with downregulated genes involved in essential physiological processes: (1) ribosome biogenesis, and (2) electron transfer and oxidative phosphorylation (Figure 7C, Supplementary Table S3). 

At least twenty-nine ribosomal structural proteins and seven enzymes involved in ribosome biogenesis resulted in being strongly downregulated upon the exposure of *H. pylori* cells to lethal concentrations of HsrA inhibitor V. In fact, some of these proteins corresponded with those DEGs with a major decrease in transcript abundance including not only the 50S ribosomal proteins L21, L35, and L27, but also the 30S ribosomal protein S10, all of which exhibited a >5.8-fold depletion in gene expression (Figure 7B,C, Supplementary Table S3). Notably, the depletion in ribosomal protein abundances was accompanied by a noticeable increase in ribosomal RNA transcription (Figure 7B,C, Supplementary Table S3). Taking into account that rRNA genes have been traditionally used as housekeeping controls in *H. pylori* due to their relatively constant rates of expression under very different environmental conditions, we hypothesized that the marked increase in rRNAs observed here could be a pleiotropic adaptive response of the cell in an attempt to overcome the lethal disruption of ribosome biogenesis. In addition, the arrest of translation as a consequence of ribosome deficit could also explain the significant increase in tRNA biosynthesis as well as in a variety of enzymes involved in amino acid metabolism. However, these hypotheses do not rule out the possibility that some of these DEGs actually constitute unrecognized or little studied direct targets of HsrA transcriptional regulation [49].

The in vivo inhibition of HsrA also led to a decrease in the transcript amounts of its previously recognized target *cyt553* [49]. Moreover, other components of the respiratory chain and coupled oxidative phosphorylation including subunit NuoN of NADH-quinone oxidoreductase, cytochrome b subunit of fumarate reductase (FrdC), and subunits A, B, B′, and D of ATP synthase also diminished their transcription (Supplementary Table S3). 

Exposure of *H. pylori* to the bactericidal HsrA inhibitor V induced the expression of antioxidant enzymes and chaperones including *katA*, *sodB*, *ccP*, *dnaK*, *groEL*, *clpB*, *grpE*, and *htpG*, among other oxidative stress response proteins (Supplementary Table S3). Notably, the *H. pylori* non-haem iron-containing ferritin (*pfr*, *hp0653*) and the ferritin-like iron-binding protein NapA appeared to be 4.7- and 2.3-fold upregulated, respectively, while the high-affinity ferrous iron transporter FeoB [57] was ~2.5-fold downregulated. Ferritin is required to sequester cytoplasmic free Fe^2+^ in order to minimize the formation of highly reactive hydroxyl radicals by the Fenton reaction, playing a major role in the protection against oxidative stress in *Campylobacterota* [58,59]. A similar free-iron removal role has been described for the neutrophil-activating protein NapA in *H. pylori* [60,61]. 

Unexpectedly, the transcriptomic analysis revealed that at least 42 genes related to *H. pylori* pathogenicity significantly changed their expression upon the in vivo inhibition of HsrA including *cag* pathogenicity island genes, flagellar proteins, adhesins, toxins, and enzymes, among others (Figure 7C, Supplementary Table S3). In addition, a plethora of genes involved in other functional categories such as carbon and lipid metabolisms, biosynthesis of vitamins and cofactors, nucleotide metabolism, transcription and translation factors, DNA replication and repair, outer membrane proteins, and membrane transporters as well as metal-resistance exhibited significant changes in their transcript abundances. 

To validate the transcriptome analysis, we evaluated the transcription levels of 10 selected genes including both upregulated and downregulated DEGs according to RNA-Seq. As shown in Figure 7D and Supplementary Table S3, all of the genes evaluated by qPCR showed the same variation trend observed in RNA-Seq upon the in vivo inhibition of HsrA.

## 3. Discussion

Infection with *H. pylori* constitutes a silent pandemic of global concern. About 50% of the world’s population is estimated to be infected with this carcinogenic bacterial pathogen [5,62], although this prevalence reaches 80% or more in several countries in the Americas, Eastern Mediterranean, and Eastern Europe [63]. *H. pylori* is considered the most important risk factor for the development of gastric cancer [7], the fifth most common malignancy and the fourth leading cause of all cancer-related deaths worldwide [64]. Despite the eradication of *H. pylori* infection significantly reducing both the incidence and mortality of gastric cancer [65,66,67], the increasing resistome accumulated by this pathogen in the last years has led to a considerable drop in the efficacy of current eradication therapies [10,68,69]. To face this concerning trend, efforts have being made to discover novel treatment strategies. 

We previously validated the essential OmpR-like orphan response regulator HsrA as an effective therapeutic target against *H. pylori* [40,41,42]. HsrA affinity-based high-throughput screening (HTS) of 1200 clinically approved drugs from Prestwick Chemical Library^®^ revealed that some highly prescribed 1,4-dihydropyridine (DHP) calcium channel blockers as well as several natural flavonoids acted as HsrA low-molecular weight ligands, leading to the inhibition of the in vitro DNA-binding activity of the regulator. Some of these potentially repurposable drugs exhibited noticeable bactericidal effects against different antibiotic-resistant strains of *H. pylori* with MICs ranging from 4 to 16 mg/L [40,41] and significantly decreased gastric colonization of *H. pylori* in mice [41]. Notably, flavonoids such as chrysin and hesperetin demonstrated strong synergistic bactericidal action against *H. pylori* in combination with clarithromycin or metronidazole, supporting the potential inclusion of these natural phytochemicals as valuable adjuvants in novel eradication therapies [40,70]. 

In the present work, we identified novel low-molecular weight inhibitors of HsrA through the screening of 12,000 drug-like compounds from the Maybridge HitFinder^TM^ chemical library. The collection is highly compliant with the Lipinski’s rule-of-five, a rule of thumb to evaluate the drug-likeness of novel potential medicines [52,53]. According to the Lipinski’s rule-of-five, compounds with a molecular weight <500 Da, a calculated logarithm of the octanol-water partition coefficient (clog *P*) < 5, number of hydrogen bond acceptors ≤ 10, and number of hydrogen bond donors ≤ 5 will have better oral bioavailability and pharmacokinetics. Thus, molecules that violate more than one of these physicochemical parameters may show problems in terms of bioavailability after oral administration. 

At least six of the novel drug-like HsrA ligands identified by the affinity-based HTS of the HitFinder^TM^ collection, denoted as I, IV, V, VIII, XI and XII, noticeably inhibited the DNA binding activity of the response regulator in vitro and demonstrated strong bactericidal activities against several *H. pylori* strains with different antibiotic resistance patterns. With MIC values ranging from 0.031 to 4 mg/L, some of these novel antimicrobial candidates constitute the most potent bactericidal HsrA inhibitors discovered to date. In addition, at least four of these highly bactericidal HsrA ligands exhibited narrow-spectrum antimicrobial activities against *H. pylori*, with MBC values ≥ 64 mg/L against several representative Gram-positive and Gram-negative members of the normal human microbiota. Since protein HsrA is unique among members of the phylum *Campylobacterota* (former class *Epsilonproteobacteria*) [45], the results suggest that certain low-molecular weight inhibitors of HsrA could specifically bind this response regulator without affecting the biological activities of essential proteins from other bacterial species. Theis evidence increases the value of HsrA as an effective and selective therapeutic target for the development of novel precision antibiotics against *H. pylori*, moving away from the risk of dysbiosis observed with the current eradication therapies [28,31]. Since the evaluation of the antimicrobial effects of our candidates on the normal microbiota presented here was limited, additional studies must be carried out in order to discern possible interactions with other commensal microbial species that could generate undesirable dysbiosis phenomena on human microbiota.

Notably, one of the novel drug-like HsrA inhibitors described here, denoted as ligand V and named N′-{[(4-chloroanilino)carbonyl]}oxy-5-nitrothiophene-3-carboximidamide in the ChemSpider database “https://www.chemspider.com (accessed on 19 September 2024)”, showed significantly higher antimicrobial activities than the rest of the compounds against all of the *H. pylori* strains used in the study. With MIC values ranging from 0.031 to 0.125 mg/L, the antimicrobial potency of ligand V was at least 4-fold higher than the second most active bactericidal HsrA inhibitor, the ligand XII, and more than 10-fold stronger than metronidazole against the same strains. In addition, ligand V demonstrated the highest therapeutic index among all highly bactericidal HsrA inhibitors described here, and showed additive antimicrobial action in combination with clarithromycin and metronidazole. 

Several factors could contribute to the higher antimicrobial potency exhibited by ligand V on *H. pylori* cells. For instance, a favorable balance among the physicochemical properties of this compound could make a difference in the microbial membrane translocation and intracellular concentration compared with other HsrA bactericidal ligands. In this context, the lipophilicity/hydrophilicity ratio, the molecular weight, the structural flexibility (rotatable bonds), number of H-bond donors and acceptors, but also the polar surface area of small molecules may greatly influence their permeability through bacterial membranes as well as their possible efflux outside the pathogen [71,72,73,74]. Although the contribution of each physicochemical property to membrane permeation and efflux varies among different bacteria species [74], it is well-recognized that the polar nature of the outer membrane of Gram-negative pathogens hinders the passive translocation of highly hydrophobic molecules [71,75]. Thus, decreasing the lipophilicity/hydrophilicity ratio of a low-molecular weight inhibitor of *E. coli* DNA gyrase by reducing the logD from 2.59 to 1.75 led to a 4-fold increase in its antibacterial potency [72]. Likewise, fine-tuning the physicochemical properties of a topoisomerase inhibitor in *Pseudomonas aeruginosa* by lowering the logD from 2.0 to 0.9 resulted in 8-fold increase in its antibacterial activity [75]. On the other hand, Gram-negative pathogens express a plethora of broad-specific efflux pumps that actively export a variety of foreign molecules out of the cell, thereby decreasing the effective antibiotic concentration in the cytoplasm [76]. Some evidence has suggested that highly polar and small compounds or very large and zwitterionic molecules are less susceptible to efflux [71]. 

Herein, time-kill kinetic studies demonstrated that ligand V and VIII produced a significantly faster decline in bacterial counts compared to the rest of the HsrA bactericidal inhibitors, suggesting similar permeability through the *H. pylori* membrane. However, the polar surface area (TPSA) of ligand V almost duplicated this property in ligand VIII, which could be linked to a higher propensity of the last compound to be expelled by *H. pylori* efflux pumps. In fact, almost 30 genes related to different efflux pumps have been identified in the *H. pylori* genome [77]. 

The antimicrobial potency of HsrA ligands could also be influenced by the binding affinity of each molecule to its target protein. According to our ITC studies, the interaction of ligand V with HsrA occurred with a >3-fold higher affinity than the interaction of ligand XII, the second most potent bactericidal compound. Despite ligand IV binding HsrA with the highest affinity, its larger molecular weight and lipophilicity could negatively affect membrane permeation. 

Aside from its potent antimicrobial activity against *H. pylori*, ligand V stood out for its strong bactericidal effect against *C. jejuni*, another clinically relevant pathogen of the phylum *Campylobacterota*. *C. jejuni* is considered the leading cause of food-borne bacterial gastroenteritis worldwide [78]. Infection in humans usually occurs through the consumption of contaminated foods (mainly poultry) or direct contact with animal hosts (including pets) and environmental reservoirs [79,80]. Despite most cases of human campylobacteriosis producing mild and self-limiting diarrhea and may not require antimicrobial therapy [78,81], the use of antibiotics could be necessary to shorten the duration of illness and to prevent severe complications in more susceptible populations including very young children, the elderly, and immunocompromised patients [78,82]. In addition, antibiotics must be used in cases of invasive or extra-gastrointestinal manifestations such as meningitis, bacteremia and endocarditis, among others [81,83,84]. 

When antibiotics are needed, the recommended first-line treatment of campylobacteriosis consists of macrolides such as azithromycin and erythromycin [85]. Fluoroquinolones like ciprofloxacin can be used as an alternative therapy, though intravenous aminoglycosides or carbapenems are recommended in severe systemic cases. Tetracyclines and chloramphenicol are also alternative therapies, but they are usually avoided in young children due to their adverse effects [82,85]. As in the case of *H. pylori*, the increasing rates of antibiotic resistance in *Campylobacter* strains isolated from humans and animals worldwide, especially to fluoroquinolones but also to macrolides [86,87,88], have prompted the WHO to include *C. jejuni* as a high priority pathogen in the search of novel antimicrobials [24]. 

Among all of the novel drug-like HsrA bactericidal inhibitors described in this work, only the ligand V exhibited a strong antimicrobial effect on *C. jejuni*, with an MIC = 0.25 mg/L. This antimicrobial potency is similar to that observed with ciprofloxacin, and stronger than those observed with doxycycline, erythromycin, and gentamicin against the same *C. jejuni* strain used herein [89]. Molecular interaction of ligand V with CosR was demonstrated by ITC analysis, while this compound noticeably inhibited the in vitro DNA binding activity of CosR according to the EMSA experiments. Therefore, the strong antimicrobial activity of ligand V on *C. jejuni* appears to rely on the inhibition of the essential biological activity of CosR [90,91,92,93], thereby validating this protein as an effective therapeutic target against *C. jejuni* [44]. The dual inhibition triggered by the same low-molecular weight ligand on the biological activities of HsrA and CosR could be expected given the high sequence identity of both ortholog proteins, especially in their C-terminal DNA binding domains (60% overall identity, 85% identity in the effector domain) [45]. 

A transcriptomic analysis was carried out in order to discern the global effects of lethal concentrations of ligand V on the *H. pylori* physiology. The results observed here appeared to correspond to the effective in vivo inhibition of HsrA and confirmed previous experimental evidence of Pelliciari and colleagues [49] about the key role of this essential response regulator in the control of protein biosynthesis and energy metabolism. The exposure of *H. pylori* to the highly bactericidal HsrA inhibitor V particularly disturbed the transcription of genes involved in ribosome biogenesis [94]. The strong detriment observed in the expression of a plethora of ribosomal proteins could certainly arrest translation and protein biosynthesis, leading to cell death [95]. In addition, the in vivo inhibition of HsrA led to a significant decrease in the transcription of several components of the respiratory chain and ATP synthase. Proteome imbalance in the electron transport chain could impair redox reactions and proton translocation across the cytoplasmic membrane, shrinking the transmembrane electrochemical proton gradient that drives the synthesis of ATP [96,97]. Furthermore, the disruption of ATP biosynthesis could additionally be exacerbated by an altered expression of ATP synthase subunits. On the other hand, imbalance in the respiratory chain may increase the production of reactive oxygen species, leading to oxidative stress [98]. This deleterious condition could induce the expression of antioxidant enzymes, heat shock proteins, and other molecular chaperones [99,100], a transcriptional response observed in this work upon the exposure of *H. pylori* to the HsrA inhibitor V. Taken together, the bactericidal effect triggered by the in vivo inhibition of the essential regulatory role of HsrA seems to be a consequence of synergistic deleterious disturbances in the *H. pylori* physiology including the arrest of protein biosynthesis, malfunction of the respiratory chain, detriment in ATP generation, and oxidative stress. The notorious increase in the transcription of a plethora of outer membrane proteins and other membrane transporters after the exposure of *H. pylori* to an HsrA inhibitor could lead to an enhancement in cell membrane permeability, which could be one of the causes of the synergistic and additive effects observed with some first-line antibiotics such as clarithromycin and metronidazole [101]. 

Due to the unsuccessful attempts to manipulate the expression of the essential *hsrA* gene in vivo [45,47], the elucidation of the HsrA regulon has represented a challenge. Previous studies have identified several dozens of putative target genes and a handful of well-characterized targets [46,47,48,49]. Our transcriptomic data suggest that HsrA acts as a global transcriptional regulator, modulating directly or indirectly the expression of up to 23% of the ORFs encoded by the *H. pylori* 26696 genome. Further analyses must be conducted in order to completely unravel the global regulatory role of HsrA and its crucial contribution to the viability and pathogenicity of *H. pylori*. 

Overall, our findings appear to suggest that the novel HsrA inhibitors described here could selectively interact with the *H. pylori* essential response regulator HsrA or its ortholog proteins in other *Campylobacterota* species, thereby acting as narrow-spectrum antimicrobials. Despite this type of antibiotic reduces the emergence and dissemination of antimicrobial resistance because it does not exert selective pressure upon non-targeted pathogens and commensal bacteria, the appearance of novel resistance mechanisms against these compounds after its translation to clinical practice could be mitigated with rational use, but cannot be avoided. Inexorably, bacteria will evolve strategies to overcome the action of antimicrobial compounds in order to survive. In fact, narrow-spectrum antibiotics already deployed in clinical practice such as fidaxomicin for the treatment of *C. difficile* infections and bedaquiline for the treatment of multidrug-resistant *Mycobacterium tuberculosis* have begun to exhibit reduced rates of resistance [102,103]. A single point mutation in HsrA that causes a change in any amino acid essential for ligand binding but not relevant for protein activity may cause the emergence of resistance to these novel antimicrobials. However, rational and personalized use of these novel narrow-spectrum antimicrobials as well as their administration into effective combinatory therapies could substantially delay this scenario.

In conclusion, the results presented here strongly support the use of HsrA and its ortholog proteins as effective and selective therapeutic targets for the development of novel antimicrobial strategies against clinically relevant pathogenic members of the phylum *Campylobacterota*. The novel drug-like highly bactericidal inhibitors of HsrA described here constitute valuable candidates for a new family of narrow-spectrum antibiotics that allow overcoming the current resistome, protecting from dysbiosis, and enhancing the battery of therapeutic options for novel personalized treatments against *H. pylori*. Translation of these findings into clinical practice will require additional preclinical studies in order to evaluate the efficacy and safety in appropriate animal models prior to clinical trials. 

## 4. Materials and Methods

### 4.1. Bacterial Strains and Culture Conditions

*H. pylori* reference strains ATCC 700392 (aka 26695), ATCC 43504 (metronidazole-resistant), and ATCC 700684 (clarithromycin-resistant) as well as *Campylobacter jejuni* ATCC 33560 were purchased from the American Type Culture Collection (Rockville, MA, USA). The *H. pylori* strain Donostia 2 (levofloxacin-resistant) was isolated from gastroduodenal biopsies and kindly donated by Dr. Milagrosa Montes from Donostia University Hospital (San Sebastian, Spain). *Helicobacter* sp. and *Campylobacter* sp. strains were routinely grown in Blood Agar Base No. 2 (OXOID, Basingstoke, UK) supplemented with 8% defibrinated horse blood (OXOID) at 37 °C for 48–72 h, under microaerobic conditions (85% N_2_, 10% CO_2_, 5% O_2_). In some determinations, bacteria were cultured in brain heart infusion broth (OXOID) supplemented with 4% fetal bovine serum (Gibco, Carlsbad, CA, USA) at 37 °C for 48–72 h under microaerobiosis. 

Reference strains Escherichia coli ATCC 25922, Klebsiella pneumonia ATCC 700603, Enterococcus faecalis ATCC 29212, Staphylococcus aureus ATCC 29213, Staphylococcus epidermidis ATCC 12228, and Streptococcus agalactiae ATCC 12386 were kindly donated by the Microbiology Service of University Clinic Hospital Lozano Blesa (Zaragoza, Spain). These strains were routinely grown in Mueller–Hilton agar/broth (PanReac AppliChem, Barcelona, Spain), overnight at 37 °C.

### 4.2. Chemicals

The Maybridge HitFinder^TM^ chemical library was purchased from Thermo Fisher Scientific (Waltham, MA, USA). All compounds in the chemical library were provided as 10 mM solutions dissolved in 100% dimethyl sulfoxide (DMSO). Selected compounds from the library were purchased in additional amounts from Thermo Fisher Scientific, dissolved in 100% DMSO, and stored at −20 °C until use. Conventional antibiotics including clarithromycin (CLR), metronidazole (MTZ), levofloxacin (LVX), and ampicillin (AMP) were purchased from Merck (Rahway, NJ, USA). Stock solutions of these drugs at 10.24 g/L in 100% DMSO were stored at −20 °C for up to 30 days.

### 4.3. Recombinant Expression and Purification of Response Regulators

The *H. pylori* 26695 HsrA protein was overexpressed in *E. coli* BL21(DE3) (EMD Biosciences) and purified by immobilized metal-affinity chromatography (IMAC) as previously described [40]. The complete coding sequence of the *cosR* gene from *C. jejuni* ATCC 33560 was amplified by PCR and cloned into the expression vector pET-28a (Novagen). Sequences of primers used for cloning *hsrA* and *cosR* are described in Supplementary Table S4. Recombinant His-tagged CosR was overexpressed in *E. coli* BL21(DE3) and purified by IMAC-Ni^2+^ using an imidazole gradient for elution. Purified protein was dialyzed in 50 mM Tris–HCl (pH 8), 300 mM NaCl, and 10% glycerol. Concentration of the purified recombinant proteins were estimated using the BCA™ Protein Assay Kit (Thermo Fisher Scientific, Bothell, WA, USA). DNA binding activities of the purified recombinant response regulators HsrA and CosR were assessed by EMSAs, as described below. 

### 4.4. High-Throughput Screening

High-throughput screening (HTS) of the Maybridge HitFinder^TM^ chemical library for *H. pylori* HsrA ligands was assessed by a fluorescence-based thermal shift assay, according to previously described procedures [40,54]. Briefly, a reaction mixture containing 50 mM Tris-HCl (pH 8), 150 mM NaCl, 10% glycerol, 5 mM DTT, SYPRO^®^ Orange ready-to-use fluorescent stain (Thermo Fisher Scientific) at a final concentration of 10 ×, and 10 µM HsrA was freshly prepared. Next, 90 μL was dispensed in every well of V-shape 96-well plates ABgene^TM^ Thermo-Fast^TM^ 96 (Thermo Fisher Scientific). Wells of columns 1 and 12 received 10 μL of DMSO (vehicle) and were used as the reference controls. The rest of the wells (columns 2 to 11) received 2.5 μL of four different compounds from the HitFinder library (250 μM as final concentration each). The unfolding curve corresponding to each well was registered from 25 °C to 75 °C in 1 °C steps using an Mx3005P*™* qPCR System (Agilent Technologies, Inc., Santa Clara, CA, USA), and analyzed by Origin 7.0 (OriginLab, Northampton, MA, USA) using a homemade script that determined the midpoint temperature of unfolding (*T*_m_). Compounds that clearly increased the thermal stability of HsrA observed in the reference controls (*T*_m_ ≥ 5 °C) were considered as HsrA ligands. 

### 4.5. Electrophoretic Mobility Shift Assays 

The DNA binding activities of recombinant HsrA and CosR were assessed in vitro by electrophoretic mobility shift assays (EMSAs). A 300-bp promoter region of the *H. pylori* 26695 operon *porGDAB* was used as the target sequence of HsrA in all EMSA experiments [48]. Likewise, a 420-bp sequence of the *C. jejuni sodB* promoter (ATCC 33560) was used as the target DNA of CosR [90]. The oligonucleotides used for the synthesis of these promoter regions are described in Supplementary Table S4. Response regulators HsrA (6 μM) or CosR (700 nM) were mixed with 120 ng of their target promoters in a reaction buffer of 10 mM bis-Tris (pH 7.5), 40 mM KCl, 100 mg/L BSA, 1 mM DTT, and 5% glycerol. For the in vitro inhibition analyses, mixtures of target DNA and response regulator were exposed to 2, 1, 0.5, and 0.1 mM of each compound of interest, incubated at room temperature for 20 min, and then separated on a 6% non-denaturing electrophoresis. DMSO instead of inhibitors was included as a vehicle control, while an internal sequence of the *Anabaena* sp. *pkn22* gene was used as the non-specific competitor in all assays. For DNA-protein binding visualization, gels were stained with the SYBR Safe DNA gel stain (Invitrogen) and analyzed with a Gel Doc 2000 Image Analyzer (Bio-Rad). 

### 4.6. Minimal Inhibitory and Bactericidal Concentrations

The antimicrobial activity of the HsrA inhibitors was evaluated by the determination of the minimal inhibitory concentration (MIC) against four different strains of *H. pylori*: ATCC 700392 (aka 26695), ATCC 43504 (MTZ-resistant reference strain), ATCC 700684 (CLR-resistant reference strain), and Donostia 2 (LVX-resistant clinical isolate). The MICs were determined by the broth microdilution method, as previously described [42]. Briefly, *H. pylori* strains were grown in blood agar at 37 °C for 72 h under microaerobic conditions. Fresh inoculums adjusted at OD_600_ = 0.01 [~10^6^ colony forming units (CFU) per mL] in BHI broth supplemented with 4% fetal bovine serum (BHI + FBS) were prepared and immediately exposed to final concentrations between 64 and 0.031 mg/L of each compound of interest. The compound solvent (DMSO) as well as conventional antibiotics (MTZ, CLR, LVX) were included as controls. MIC values were determined colorimetrically after 48 h at 37 °C by using resazurin. For the minimal bactericidal concentration (MBC) determinations, aliquots from MIC plates were aseptically seeded on blood agar and incubated for 72 h at 37 °C under microaerobic conditions. Each experiment was performed twice in triplicate. The MIC and MBC values of the selected compounds against *C. jejuni* ATCC 33560 were determined by using the same method and culture conditions described for the *H. pylori* strains.

The antimicrobial activities of the selected compounds against several Gram-negative and Gran-positive representative species of normal human microbiota were determined according to the EUCAST guidelines [104]. Briefly, standardized inoculums (0.5 McFarland) were freshly prepared from overnight Mueller–Hinton agar plates of reference strains *E. coli* ATCC 25922, *K. pneumonia* ATCC 700603, *E. faecalis* ATCC 29212, *S. aureus* ATCC 29213, *S. epidermidis* ATCC 12228, and *S. agalactiae* ATCC 12386. Next, bacterial suspensions adjusted to 5 × 10^5^ CFU/mL in Mueller–Hinton broth were exposed to a range of concentrations from 64 to 0.031 mg/L of the selected compounds. DMSO and conventional antibiotics LVX and AMP were included as controls. The MBCs were determined by seeding aliquots from dilutions around the MIC values on Mueller–Hinton agar plates, which were incubated for 24–48 h at 37 °C. Experiments were performed twice in triplicate. 

### 4.7. Time-Kill Kinetic Assays

Time-kill kinetics of selected HsrA inhibitors were carried out as previously described [42], with slight modifications. Briefly, fresh grown cells of *H. pylori* strain ATCC 700392 were resuspended in BHI + FBS at 1.0 × 10^4^ CFU/mL and exposed to 4 × MIC of each compound. Bacteria exposed to DMSO (vehicle) instead of the HsrA inhibitors were included as a control. All cultures were incubated at 37 °C under microaerobic conditions, and aliquots were taken after 0, 2, 4, 8, and 24 h of exposure for CFU determination. Experiments were performed twice in triplicate. Results were presented as log_10_ CFU/mL versus hours of exposure. Differences in *H. pylori* survival were subjected to statistical analysis by using the Mann–Whitney *U* test.

### 4.8. Checkerboard Assays

Potential synergisms between the selected HsrA inhibitors and first-line antibiotics were evaluated by using checkerboard assays, as previous described [40,42]. Briefly, the antimicrobials to be tested were twofold serially diluted in BHI + FBS using independent sterile microtiter plates. Then, concentration gradients from two antimicrobials were aseptically mixed at equal volumes in a new microtiter plate, resulting in a matrix of 77 unique combinations of both compounds. Next, freshly prepared inoculum of *H. pylori* ATCC 700392 adjusted at 2 × 10^6^ CFU/mL in BHI+ FBS were added to all wells, and plates were incubated for 72 h at 37 °C under microaerophilic conditions. Microbial growth was revealed by adding filter-sterilized resazurin up to 0.01 mg/mL and further incubation for 6 h. The fractional inhibitory concentration index (FICI) for each antimicrobial combination was calculated as previously described. Values of FICI ≤ 0.5, 0.5 < FICI ≤ 1, 1 < FICI ≤ 4, and FICI > 4 were considered as indicative of synergistic, additive, neutral, or antagonist interactions, respectively [40,42].

### 4.9. In Vivo Inhibition of HsrA

Bacterial growth from three blood agar plates of *H. pylori* ATCC 700392 (aka 26695) incubated for 48 h at 37 °C under microaerobic conditions was aseptically resuspended in BHI + FBS and adjusted to 10^7^ CFU/mL. BHI broth cultures were incubated microaerobically at 37 °C for 24 h. Next, aliquots of 12 mL were exposed in triplicate to 4 × MIC of HsrA inhibitor V and further incubated in the same above conditions until the log_10_ CFU/mL resulted in being diminished in one unit. At this time, bacterial growth was stopped by adding 1.5 mL ice-cold RNA stop solution (95% absolute ethanol, 5% acid-buffered phenol) and subsequently incubated on ice. Control samples were exposed to the same amount of DMSO instead of the inhibitor. *Helicobacter* cells from all samples were harvested by centrifugation (4000 rpm at 4 °C for 10 min), then the pellets were quickly frozen by immersion in liquid nitrogen and stored at −80 °C. 

### 4.10. RNA Sequencing

RNA-Seq analysis was performed by GENEWIZ (Azenta Life Sciences, Leipzig, Germany). Frozen cell pellets from two biological replicates of each treatment condition were properly submitted to GENEWIZ in dry ice. RNA-Seq libraries were prepared according to standard protocols via rRNA depletion, RNA fragmentation and random priming, reverse transcription, 5’-phosphorylation and dA-tailing of cDNAs, adaptor ligation, and PCR enrichment. Once the quality of each library was confirmed, they were sequenced on an Illumina NovaSeq 6000 (Illumina, San Diego, CA, USA) with 150-bp paired-ends. Raw sequence reads were filtered to remove possible adapter sequences and nucleotides with poor quality using Trimmomatic v.0.36 [105]. The trimmed reads were subsequently mapped to the *H. pylori* strain 26695 reference genome NC_000915.1 available on NCBI, by using the Bowtie2 aligner v.2.2.6 [106]. Unique gene hit counts were calculated by using featureCounts [107] from the Subread package v.1.5.2. Only unique reads that fell within the gene regions were counted. Using DESeq2 [108], a comparison of the gene expression between the control and treated groups of samples was performed. The Wald test was used to generate *p*-values and log2 fold changes. Genes with an adjusted *p*-value < 0.05 and absolute log2 fold change >1 were considered as differentially expressed genes (DEGs) for each comparison. The RNA-Seq data generated in this study have been deposited in ArrayExpress with the accession number E-MTAB-14255.

### 4.11. Quantitative Real-Time PCR 

For the RNA extraction for the qPCR analyses, frozen pellets were quickly resuspended in 850 μL lysis buffer containing 20 mM sodium acetate (pH 5.2), 0.5% SDS, and 1 mM Na_2_-EDTA. Then, 850 μL of acid phenol preheated at 65 °C was immediately added to the cell suspensions. Samples were vigorously mixed by vortex and incubated at 65 °C for 10 min. Cell extracts were centrifuged at 13,000 rpm for 5 min at room temperature and the aqueous phase was transferred to clean RNase-free tubes. Next, samples were treated with 1 mL of TRIzol^TM^ Reagent (Thermo Fisher Scientific) for 5 min, after which 200 μL of chloroform was added and vigorously mixed. The colorless upper aqueous phase was sequentially washed three times with 1 V of chloroform, and the total RNA was precipitated overnight at −80 °C with 2.5 V of ice-cold absolute ethanol. The RNA samples were resuspended in 50 μL RNase-free water, and genomic DNA was subsequently removed by using the TURBO DNA-Free™ Kit (Thermo Fisher Scientific). The purity and integrity of the RNA samples were checked using both a NanoDrop spectrophotometer (Thermo Fisher Scientific) and electrophoresis. The absence of residual DNA was determined by qPCR. 

Reverse transcription was carried out with SuperScript retrotranscriptase (Invitrogen, Waltham, MA, USA) in a reaction buffer containing 150 ng of random primers (Invitrogen), 1 mM dNTP mix (GE Healthcare, Tokyo, Japan), and 10 mM DTT. Quantitative PCRs were performed by using a QuantStudio™ 5 Real-Time PCR System (Applied Biosystems, Waltham, MA, USA) in 30 μL of reaction mixtures containing 10 ng of cDNA, 12.5 μL of SYBR Green PCR Master Mix (Thermo Fisher Scientific), and 0.4 μL of primers at 25 μM in water. The sequences of primers used for each gene are described in Supplementary Table S4. Relative quantification was performed according to the ΔΔCt method [109]. Expression levels were normalized using the *H. pylori* 26695 *glnA* gene as housekeeping [110].

### 4.12. Cytotoxicity and Therapeutic Index

The in vitro toxicity of the selected HsrA inhibitors was determined on HeLa cells by the PrestoBlue^TM^ assay, as previously described [42]. Briefly, cells were cultured at 37 °C with 5% CO_2_ in Dulbecco’s modified Eagle’s medium containing 10% fetal bovine serum, 1% L-GlutaMAX^TM^ solution (Thermo Fisher Scientific), and 1% penicillin/streptomycin. When 80% confluence was achieved, cells were detached, counted, seeded in 96-well microplates at a density of 10,000 cells per well, and allowed to adhere for 24 h. Next, the mammalian cells were exposed to selected compounds at a range of 0.125 to 128 mg/L. After 24 h of exposure, the cell viability was measured with the PrestoBlue^TM^ cell viability reagent (Thermo Fisher Scientific), according to the manufacturer’s instructions. Experiments were performed twice in triplicate. The 50% cytotoxic concentration (CC_50_) of each compound was calculated by regression analysis using Microsoft Excel. Therapeutic index (TI) values were calculated as the ratio of the CC_50_ to the higher MIC observed for each compound [111]. 

### 4.13. Isothermal Titration Calorimetry

Molecular interactions of the selected ligands with proteins HsrA or CosR were studied by isothermal titration calorimetry (ITC), as previously described [42]. Titrations were assessed in a high-sensitivity MicroCal Auto-iTC200 calorimeter (Malvern Panalytical, Malvern, UK). Experiments were carried out at 25 °C in freshly prepared buffer containing 50 mM Tris-HCl [pH 8], 150 mM NaCl, 10% glycerol, and 1% DMSO. A 20 µM solution of protein was located in the calorimetric cell. Then, a sequence of 19 injections of 2 µL volume each with a 200 µM solution of the corresponding ligand was programmed with a time spacing of 150 s, a stirring speed of 750 rpm, and a reference power of 10 µcal/s in the sample cell. Thermodynamic properties such as binding stoichiometry, dissociation constants, and binding enthalpies were calculated by nonlinear least-squares regression data analysis using Origin 7.0 software. 

### 4.14. Molecular Docking 

Predicted models of interaction between the response regulator HsrA and selected compounds were determined by molecular docking analyses using AutoDock Vina “vina.scripps.edu (accessed on 19 September 2024)” [112]. The 3D structures of the compounds were built in Corina Classic “www.mn-am.com (accessed on 19 September 2024)” and energy was minimized using the web server AMMOS2 [113]. The 3D structure of HsrA (2HQR, model 1, chain A) was obtained from the Protein Data Bank. The protein structure was considered rigid, while ligands were treated as flexible molecules with free rotatable bonds. Interaction energy was estimated by AutoGrid4 [114] and the top-ranked pose for each ligand was considered as the predicted model of interaction. Protein-ligand complexes were visualized by PyMOL “www.pymol.org (accessed on 19 September 2024)”.

## 5. Patents

The authors declare that a patent has been filed concerning the use of compounds for the treatment and/or prevention of an infection or disease caused by *Helicobacter* or *Campylobacter*.

## Figures and Tables

**Table 1:** Chemical structure and physicochemical properties of relevant HsrA ligands identified by the fluorescent thermal shift-based high-throughput screening (HTS) of the Maybridge HitFinder^TM^ chemical library.

Ligand	Chemical Structure	Formula	Molecular Weight (Da)	Log *P* ^1^	H-Bond Donors	H-Bond Acceptors	Lipinski Violations	TPSA (Å) ^2^	Rotatable Bonds	Log *D* ^3^
I	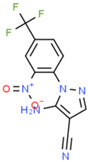	C_11_H_6_F_3_N_5_O_2_	297.19	1.56	1	7	0	113.45	3	2.26
IV	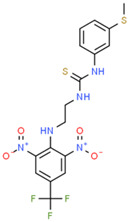	C_17_H_16_F_3_N_5_O_4_S_2_	475.47	3.08	3	7	0	185.12	11	4.97
V	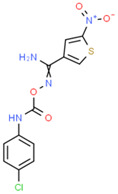	C_12_H_9_ClN_4_O_4_S	340.74	1.99	2	5	0	150.77	6	2.92
VIII	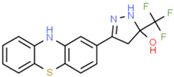	C_16_H_12_F_3_N_3_OS	351.35	3.50	3	5	0	81.95	2	3.60
XI	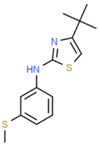	C_14_H_18_N_2_S_2_	278.44	4.18	1	1	0	78.46	4	4.77
XII	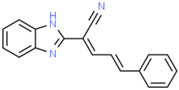	C_18_H_13_N_3_	271.32	3.53	1	2	0	52.47	3	3.79

Molecular properties obtained from the SwissADME web tool “http://www.swissadme.ch/ (accessed on 19 September 2024)” and the ChemSpider database “www.chemspider.com (accessed on 19 September 2024)”. ^1^ Log *P* values correspond to consensus Log *P*_o/w_ from SwissADME. ^2^ TPSA, topological polar surface area. ^3^ Log *D* values correspond to ACD/LogD (pH 7.4) from ChemSpider.

**Table 2:** Minimal inhibitory and bactericidal concentrations of HsrA inhibitors against different strains of *H. pylori*.

Inhibitor or Drug	MIC (MBC), mg/L			
	* H. pylori * ATCC 700392 (26695)	* H. pylori * ATCC 43504 (MTZ-R)	* H. pylori * ATCC 700684 (CLR-R)	* H. pylori * Donostia 2 (LVX-R)
I	4 (4)	1 (1)	4 (4)	2 (2)
IV	2 (4)	2 (4)	4 (4)	4 (4)
V	0.063 (0.125)	0.125 (0.125)	0.031 (0.031)	0.125 (0.125)
VIII	1 (2)	1 (2)	1 (2)	2 (2)
IX	8 (16)	4 (8)	4 (8)	8 (16)
X	8 (16)	8 (8)	8 (8)	8 (16)
XI	4 (4)	2 (4)	2 (4)	2 (4)
XII	2 (2)	0.5 (0.5)	0.5 (0.5)	1 (1)
XVII	16 (16)	4 (8)	8 (8)	8 (8)
XXVI	16 (16)	8 (8)	8 (8)	8 (16)
XXVIII	32 (64)	16 (16)	32 (32)	32 (32)
XXXI	>64 (>64)	64 (64)	>64 (>64)	>64 (>64)
MTZ	1 (2)	64 (128)	1 (2)	8 (8)
CLR	<0.03 (<0.03)	<0.03 (<0.03)	16 (32)	<0.03 (<0.03)
LVX	0.12 (0.12)	0.5 (0.5)	0.12 (0.12)	16 (32)

MTZ-R, metronidazole-resistant strain. CLR-R, clarithromycin-resistant strain. LVX-R, levofloxacin-resistant strain. MIC, minimal inhibitory concentration. MBC, minimal bactericidal concentration.

**Table 3:** Interaction of selected bactericidal HsrA inhibitors with first-line antibiotics against *H. pylori* ATCC 700392.

Antibiotic ^1^	HsrA Inhibitor	FIC_antibiotic_	FIC_inhibitor_	FICI ^2^	Interaction ^3^
CLR	I	1	1	2	Neutral
	IV	1	1	2	Neutral
	V	0.5	0.06	0.56	Additive
	VIII	0.5	0.03	0.53	Additive
	XI	0.25	0.5	0.75	Additive
	XII	0.25	0.5	0.75	Additive
MTZ	I	0.125	0.5	0.625	Additive
	IV	1	1	2	Neutral
	V	0.125	0.5	0.625	Additive
	VIII	0.5	0.125	0.625	Additive
	XI	0.5	0.063	0.563	Additive
	XII	0.25	0.25	0.5	Synergism
LVX	I	1	1	2	Neutral
	IV	1	1	2	Neutral
	V	1	1	2	Neutral
	VIII	1	1	2	Neutral
	XI	1	1	2	Neutral
	XII	0.5	0.5	1	Neutral

^1^ CLR, clarithromycin. MTZ, metronidazole. LVX, levofloxacin. ^2^ The fractional inhibitory concentration index (FICI) is calculated as: FIC_A_ (MIC_A_ in the presence of B/MIC_A_ alone) + FIC_B_ (MIC_B_ in the presence of A/MIC_B_ alone). ^3^ According to the FICI value, the interaction between two compounds against a particular bacterial strain can be classified as synergism (FICI ≤ 0.5), additive (FICI > 0.5 to ≤1), no interaction or neutral (FICI > 1 to ≤4), and antagonism (FICI > 4). FIC, fractional inhibitory concentration. MIC, minimal inhibitory concentration. A and B refer to antimicrobial A and antimicrobial B.

**Table 4:** Antimicrobial activities of relevant HsrA inhibitors against *C. jejuni* and several representative members of normal human microbiota.

Inhibitor or Drug	MIC (MBC), mg/L						
	*C. jejuni* ATCC 33560	*E. coli* ATCC 25922	* K. pneumoniae * ATCC 700603	*E. faecalis* ATCC 29212	*S. aureus* ATCC 29213	* S. epidermidis * ATCC 12228	* S. agalactiae * ATCC 12386
I	32 (32)	>64 (>64)	>64 (>64)	>64 (>64)	>64 (>64)	>64 (>64)	32 (64)
IV	64 (>64)	>64 (>64)	>64 (>64)	>64 (>64)	>64 (>64)	>64 (>64)	>64 (>64)
V	0.25 (0.5)	>64 (>64)	>64 (>64)	>64 (>64)	>64 (>64)	>64 (>64)	>64 (>64)
VIII	16 (32)	>64 (>64)	>64 (>64)	64 (64)	16 (32)	4 (4)	16 (32)
XI	>64 (>64)	>64 (>64)	>64 (>64)	64 (64)	16 (32)	16 (64)	8 (8)
XII	64 (>64)	>64 (>64)	>64 (>64)	>64 (>64)	>64 (>64)	>64 (>64)	>64 (>64)
LVX	<0.12 (<0.12)	N.D.	0.5 (0.5)	N.D.	N.D.	N.D.	N.D.
AMP	N.D.	4 (4)	N.D.	4 (4)	2 (4)	4 (4)	0.25 (0.5)

LVX, levofloxacin. AMP, ampicillin. MIC, minimal inhibitory concentration. MBC, minimal bactericidal concentration.

**Table 5:** Thermodynamic parameters and interacting amino acid residues of selected HsrA-inhibitor complexes, according to isothermal titration calorimetry (ITC) and molecular docking analyses.

Inhibitor	ITC ^1^				Molecular Docking ^2^
	n	*K*_d_ (μM)	Δ*H* (kcal/mol)	Δ*G* (kcal/mol)	Interacting Residues
I	1.0	13	–1.2	–6.7	I121, I123, L126, I135, L152, L155, A156, R159, **M195**, **L199**, T203
IV	0.9	3.9	–1.6	–7.4	I135, V142, V144, K145, G146, P148, L152, **K194**, **P198**
V	1.0	13	–1.9	–6.7	L126, I135, L152, **K194**, **M195**, **P198**, **L199**, T203
VIII	0.9	10	0.8	–6.8	V144, K145, G146, F149, L152, **I191**, **K194**, **M195**
XI	1.1	49	–3.1	–5.9	I123, I128, L152, R159, D160, **M195**, **L199**, T203, C215, Y216
XII	1.0	43	0.8	–6.0	I123, L126, I128, I135, L152, **M195**, **P198**, **L199**, T203

^1^ n, stoichiometry. *K*_d_, dissociation constant. Δ*H*, enthalpy. Δ*G*, free energy. Relative error in *K*_d_ is 15%, absolute error in Δ*H* is 0.4 kcal/mol, absolute error in Δ*G* is 0.1 kcal/mol. ^2^ Amino acid residues directly involved in forming the helix-turn-helix (HTH) DNA binding motif of HsrA are highlighted in bold.

## Data Availability

The RNA-Seq data have been deposited in ArrayExpress under accession number E-MTAB-14255, The rest of the data supporting the findings of this study are available within the paper and the Supplementary Materials Files.

## Supplementary Materials

The supporting information can be downloaded at https://www.mdpi.com/article/10.3390/ijms251810175/s1.
