# Supplementary material for: Novel Drug-like HsrA Inhibitors Exhibit Potent Narrow-Spectrum Antimicrobial Activities against Helicobacter pylori
Source: Int J Mol Sci. 2024 Sep 22;25(18):10175. doi: 10.3390/ijms251810175 (PMC11432330; doi:10.3390/ijms251810175)
Supplement: Supplementary file 1 [file ijms-25-10175-s001.zip › ijms-3215682-Supplementary Materials.pdf]

Novel drug-like HsrA inhibitors exhibit potent narrow-spectrum antimicrobial activities against *Helicobacter pylori*

Javier Casado, Irene Olivan-Muro, Sonia Algarate, Eduardo Chueca, Sandra Salillas, Adrián Velázquez-Campoy, Elena Piazuelo, María F. Fillat, Javier Sancho, Ángel Lanas and Andrés González *.

*Corresponding author:
Andrés González
E-mail: andresg@unizar.es

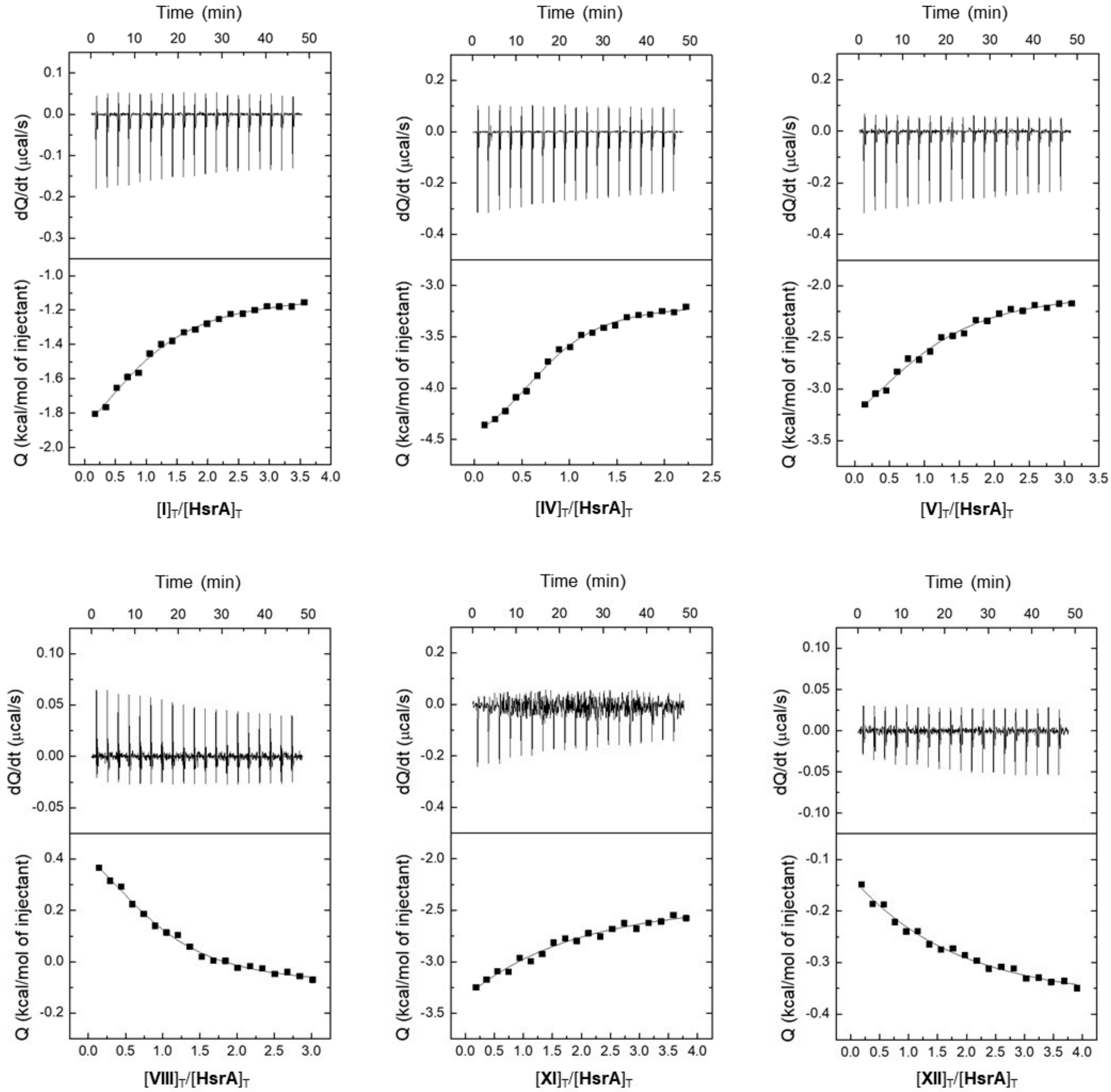

Figure S1. Isothermal titration calorimetry (ITC) analyses of the interaction between the *H. pylori* response regulator HsrA and its low-molecular weight ligands I, IV, V, VIII, XI and XII. Thermograms of the protein interaction (thermal power as a function of time) with each ligand are depicted in the upper panel of the pictures, and the respective binding isotherms (titrant normalised heat effects as a function of the ligand:protein molar ratio in the cell) are showed in the lower panels.

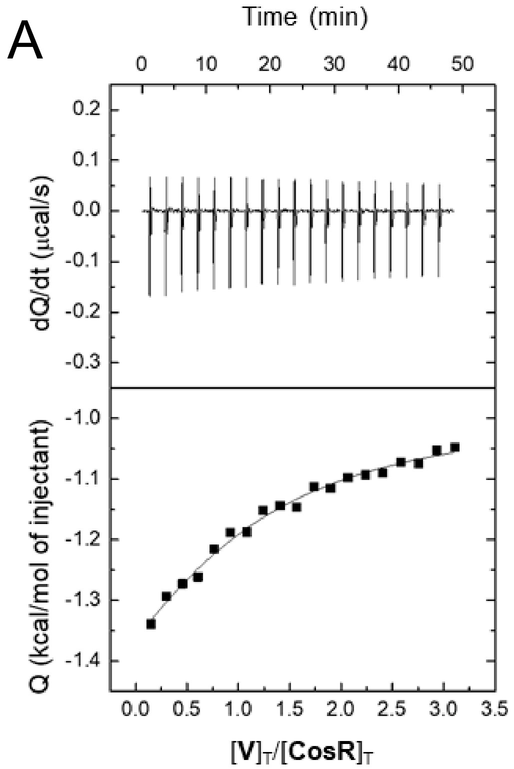

B

**Thermodynamic parameters**	
n	0.9
K_d	26 μM
ΔH	-0.9 kcal/mol
ΔG	-6.2 kcal/mol

Figure S2. ITC analysis of the interaction between the *C. jejuni* response regulator CosR and compound V. **(A)** Upper panel shows the thermogram, while lower panel shows the binding isotherm. **(B)** Thermodynamics parameters of the interaction. Relative error in K_d is 15%, absolute error in ΔH is 0.4 kcal/mol, absolute error in ΔG is 0.1 kcal/mol.

Table S1. Compounds from the Maybridge HitFinder^{TM} chemical collection identified as HsrA ligands according the fluorescent thermal shift-based HTS.

Ligand ID	Compound name	Maybridge Code	ΔT_m (°C) [1]	Molecular formula	Molecular weight	ChemSpider ID
I	5-amino-1-[2-nitro-4-(trifluoromethyl)phenyl]-1H-pyrazole-4-carbonitrile	BTB02343	38	$C_{11}H_6F_3N_5O_2$	297.19	2076080
II	3-[(4-chlorophenyl)thio]-2-methyl-1H-indole	KM06044	20	$C_{15}H_{12}ClNS$	273.78	2099536
III	3-(4-fluorophenyl)-2-(1H-indol-3-yl)acrylonitrile	PD00112	17	$C_{17}H_{11}FN_2$	262.28	2104096
IV	N-{2-[2,6-dinitro-4-(trifluoromethyl)anilino]ethyl}-N'-[3-(methylsulfanyl)phenyl]thiourea	CD03711	16	$C_{17}H_{16}F_3N_5O_4S_2$	475.47	2082455
V	N'-{[(4-chloroanilino)carbonyl]oxy}-5-nitrothiophene-3-carboximidamide	DFP00026	16	$C_{12}H_9ClN_4O_4S$	340.74	2084758
VI	N'-({[3-(trifluoromethyl)anilino]carbonyl}oxy)-2,3-dihydro-1,4-benzodioxine-2-carboximidamide	KM06580	15	$C_{17}H_{14}F_3N_3O_4$	381.3	2099748
VII	2-methyl-4-{2-[3-(trifluoromethyl)phenyl]diaz-1-enyl}aniline	BTB06669	15	$C_{14}H_{12}F_3N_3$	279.26	2078541
VIII	3-(10H-phenothiazin-2-yl)-5-(trifluoromethyl)-4,5-dihydro-1H-pyrazol-5-ol	SP01458	14	$C_{16}H_{12}F_3N_3OS$	351.35	2024533
IX	2-[3-(trifluoromethyl)-1H-pyrazol-1-yl]-N'-[({4-[(trifluoromethyl)thio]anilino}carbonyl)oxy]ethanimidamide	HAN00349	13	$C_{14}H_{11}F_6N_5O_2S$	427.33	2087678
X	2-[2-(2-methyl-1-benzofuran-3-yl)acetyl]-N-[3-(trifluoromethyl)phenyl]-1-hydrazinecarbothioamide	FV00183	11	$C_{19}H_{16}F_3N_3O_2S$	407.4	2086022
XI	N2-[3-(methylthio)phenyl]-4-(tert-butyl)-1,3-thiazol-2-amine	BTB04902	11	$C_{14}H_{18}N_2S_2$	278.44	2077472
XII	2-(1H-benzimidazol-2-yl)-5-phenylpenta-2,4-dienenitrile	PHG00723	9	$C_{18}H_{13}N_3$	271.32	606452
XIII	1-(5-isopropyl-3,8-dimethyl-1-azulenyl)-2-(4-methoxyphenyl)-1-diazene	SEW04851	9	$C_{22}H_{24}N_2O$	332.44	17921592
XIV	[1,1'-biphenyl]-4-yl(5-nitro-1-benzofuran-2-yl)methanol	RH01805	9	$C_{21}H_{15}NO_4$	345.35	2010255
XV	5-nitrothiophene-3-carbaldehyde N-(4-bromo-3-chlorophenyl)hydrazone	DFP00003	9	$C_{11}H_7BrClN_3O_2S$	360.61	7857708
XVI	1-(4-fluorophenyl)-5-methyl-N-{2-[2-nitro-4-(trifluoromethyl)anilino]ethyl}-1H-pyrazole-4-carboxamide	HTS05759	8	$C_{20}H_{17}F_4N_5O_3$	451.37	2091051
XVII	ethyl 4-[5-(4-morpholinobuta-1,3-dienyl)-2H-1,2,3,4-tetraazol-2-yl]benzoate	BTB12773	8	$C_{18}H_{21}N_5O_3$	355.39	4651038
XVIII	2-(4-chlorophenyl)-5-{[(4-pyridin-3-ylpyrimidin-2-yl)thio]methyl}-2,4-dihydro-3H-pyrazol-3-one	MWP01044	7	$C_{19}H_{14}ClN_5OS$	395.87	2102650
XIX	N1-(3-chlorophenyl)-2-{2-[(2-methyl-1H-indol-3-yl)thio]acetyl}hydrazine-1-carboxamide	KM07954	7	$C_{18}H_{17}ClN_4O_2S$	388.87	2100308
XX	4-amino-3,5-dichloro-N'-({[4-(trifluoromethoxy)anilino]carbonyl}oxy)benzenecarboximidamide	SEW03768	7	$C_{15}H_{11}Cl_2F_3N_4O_3$	423.17	2022070
XXI	2-amino-6-(tert-pentyl)-4,5,6,7-tetrahydro-1-benzothiophene-3-carbonitrile	HTS05438	7	$C_{14}H_{20}N_2S$	248.39	514469

XXII	ethyl 1-[(3-chloroanilino)carbonyl]-4-(phenylthio)-1H-pyrazole-5-carboxylate	SPB03702	7	$C_{19}H_{16}ClN_3O_3S$	401.87	2026372
XXIII	N-(1-methyl-3-phenyl-1H-pyrazol-5-yl)-N'-(2-thienyl)urea	HTS10230	7	$C_{15}H_{14}N_4OS$	298.36	2093643
XXIV	13H-dibenzo[a,i]carbazole	JFD03891	7	$C_{20}H_{13}N$	267.32	8845
XXV	N-(2-thienyl)-N'-{6-[3-(trifluoromethyl)phenoxy]-3-pyridinyl}urea	HTS08492	7	$C_{17}H_{12}F_3N_3O_2S$	379.36	2092650
XXVI	2-({6-[(2-aminophenyl)sulfanyl]-5-nitro-2-pyridyl}sulfanyl)aniline	SEW05581	6	$C_{17}H_{14}N_4O_2S$	338.38	2022927
XXVII	2-acetyl-4-nitroindane-1,3-dione	JFD02083	6	$C_{11}H_7NO_5$	233.18	2016957
XXVIII	N3-[4-(trifluoromethoxy)phenyl]-4-hydroxy-1,5,6-trimethyl-2,2-dioxo-1,2-dihydro-2lambda~6~-thieno[2,3-c][1,2]thiazine-3-carboxamide	KM04706	6	$C_{17}H_{15}F_3N_2O_5S_2$	448.44	17926070
XXIX	N-(5-cyclopropyl-1,3,4-thiadiazol-2-yl)-2-(2-fluorophenyl)acetamide	HTS02730	6	$C_{13}H_{12}FN_3OS$	277.32	2089264
XXX	N1-[5-(4-fluoroanilino)penta-2,4-dienylidene]-4-fluoroaniline hydrochloride	NRB00096	6	$C_{17}H_{14}F_2N_2.HCl$	320.76	4653283
XXXI	N-[2-(4-chlorophenyl)-3-oxo-6-(2-thienylcarbonyl)-2,3-dihydro-4-pyridazinyl]acetamide	HTS08477	5	$C_{17}H_{12}ClN_3O_3S$	373.81	2092639

[1]Increase in the T_m value of protein-ligand complex with respect to the mean T_m value of controls (protein + DMSO).

Table S3. Functional classification of DEGs

ORF	Gene name	Gene annotation[1]	Fold Change	Effect
Ribosome biogenesis				
hp0296	*rplU*	50S ribosomal protein L21	-8,68	down
hp0125	*rpmI*	50S ribosomal protein L35	-6,96	down
hp1320	*rpsJ*	30S ribosomal protein S10	-6,14	down
hp0297	*rpmA*	50S ribosomal protein L27	-5,89	down
hp0247	*deaD*	ATP-dependent RNA helicase DeaD	-4,71	down
hp1297	*rpmJ*	50S ribosomal protein L36	-4,58	down
hp0200	*rpmF*	50S ribosomal protein L32	-4,52	down
hp1149	*rimM*	16S rRNA processing protein RimM	-4,26	down
hp1040	*rpsO*	30S ribosomal protein S15	-4,08	down
hp0126	*rplT*	50S ribosomal protein L20	-4,08	down
hp0491	*rpmB*	50S ribosomal subunit protein L28	-3,87	down
hp1447	*rpmH*	50S ribosomal protein L34	-3,68	down
hp1063	*rsmG*	16S rRNA methyltransferase	-3,33	down
hp1296	*rpsM*	30S ribosomal protein S13	-3,25	down
hp1147	*rplS*	50S ribosomal protein L19	-3,13	down
hp0084	*rplM*	50S ribosomal protein L13	-3,07	down
hp1496	*rplY*	50S ribosomal protein L25	-3,05	down
hp1151	*rpsP*	30S ribosomal protein S16	-3,01	down
hp1200	*rplJ*	50S ribosomal subunit protein L10	-2,95	down
hp1428	*rlmM*	Ribosomal RNA large subunit methyltransferase N	-2,85	down
hp1303	*rplR*	50S ribosomal protein L18	-2,67	down
hp1160	*ybeY*	rRNA maturation factor	-2,56	down
hp0076	*rpsT*	30S ribosomal protein S20	-2,53	down
hp1302	*rpsE*	30S ribosomal protein S5	-2,47	down
hp0551	*rpmE*	50S ribosomal protein L31	-2,47	down
hp0399	*rpsA*	30S ribosomal protein S1	-2,45	down
hp0562	*rpsU*	30S ribosomal protein S21	-2,34	down
hp1311	*rpmC*	50S ribosomal protein L29	-2,25	down
hp1197	*rpsL*	30S ribosomal protein S12	-2,20	down
hp1319	*rplC*	50S ribosomal protein L3	-2,20	down
hp1295	*rpsK*	30S ribosomal protein S11	-2,19	down
hp0553	*rlmB*	23S rRNA (guanosine(2251)-2'-O)-methyltransferase	-2,16	down
hp0956	*rluC*	23S rRNA pseudouridine synthase	-2,13	down
hp1068	*prmA*	Ribosomal protein L11 methyltransferase	-2,10	down
hp1202	*rplK*	50S ribosomal protein L11	-2,07	down
hp1309	*rplN*	50S ribosomal protein L14	-2,01	down
hpr07	*hprrnB16S*	16S rRNA	15,28	up
hpr01	*hprrnA23S*	23S rRNA	8,08	up
hpr06	*hprrnB23S*	23S rRNA	7,82	up

hpr04	*hprrnA16S*	16S rRNA	6,23	up
hpr03	*hprrnC5S*	5S rRNA	6,00	up
hpr02	*hprrnA5S*	5S rRNA	5,86	up
hpr05	*hprrnB5S*	5S rRNA	4,07	up
hp0569	*ychF*	Ribosome-binding ATPase	3,62	up
Amino acid metabolism				
hp0695	*hyuA*	hydantoinase/oxoprolinase family protein	4,96	up
hp0696		N-methylhydantoinase	4,92	up
hp0943	*dadA*	D-amino-acid dehydrogenase	4,73	up
hp0294	*amiE*	aliphatic amidase	3,23	up
hp1398	*ald*	alanine dehydrogenase	3,15	up
hp0723	*ansB*	L - asparaginase II	3,08	up
hp1210	*cysE*	serine O-acetyltransferase	2,95	up
hp0330	*ilvC*	ketol-acid reductoisomerase	2,90	up
hp0944		2-iminobutanoate/2-iminopropanoate deaminase	2,82	up
hp0691	*yxjD*	3-oxoadipate coA-transferase subunit A	2,80	up
hp1468	*ilvE*	branched-chain amino acid aminotransferase	2,76	up
hp0510	*dapB*	4-hydroxy-tetrahydrodipicolinate reductase	2,74	up
hp0626	*dapD*	tetrahydrodipicolinate N-succinyltransferase	2,63	up
hp0020	*nspC*	carboxynorspermidine decarboxylase	2,61	up
hp0692	*yxjE*	3-oxoadipate coA-transferase subunit B	2,51	up
hp0649	*aspA*	aspartate ammonia-lyase	2,42	up
hp0056		delta-1-pyrroline-5-carboxylate dehydrogenase	2,24	up
hp0134	*dhs1*	3-deoxy-7-phosphoheptulonate synthase	2,22	up
hp0096		phosphoglycerate dehydrogenase	2,17	up
hp0663	*aroC*	chorismate synthase	2,16	up
hp1050	*thrB*	homoserine kinase	2,12	up
hp0132	*sdaA*	L-serine deaminase	2,12	up
hp0357		3-hydroxy acid dehydrogenase / malonic semialdehyde reductase	2,04	up
hp0380	*gdhA*	Glutamate dehydrogenase	2,03	up
hp0098	*thrC*	threonine synthase	-2,31	down
hp1282	*trpE*	anthranilate synthase component I	-2,12	down
hp0307	*argJ*	Arginine biosynthesis bifunctional protein ArgJ	-2,11	down
Carbon metabolism				
hp0779	*acnB*	aconitase B	4,11	up
hp1100	*edd*	phosphogluconate dehydratase	4,07	up
hp1099	*eda*	bifunctional 4-hydroxy-2-oxoglutarate aldolase/2-dehydro-3-deoxy-phosphogluconate aldolase	3,73	up
hp0697		acetone carboxylase, gamma subunit	3,43	up
hp1045	*acoE*	acetyl-CoA synthetase	3,11	up
hp1103	*glk*	glucokinase	2,83	up
hp1101	*zwf*	glucose-6-phosphate dehydrogenase	2,82	up

hp1166	*pgi*	glucose-6-phosphate isomerase	2,68	up
hp1102	*devB*	6-phosphogluconolactonase	2,50	up
hp1345	*pgk*	phosphoglycerate kinase	2,12	up
hp1104		($NADP^{+}$)-dependent alcohol dehydrogenase	2,12	up
hp0574	*lacA*	ribose 5-phosphate isomerase B	-4,10	down
hp0588	*oorD*	2-oxoglutarate ferredoxin oxidoreductase, subunit delta	-3,05	down
Lipid metabolism				
hp0557	*accA*	acetyl-CoA carboxylase carboxyl transferase subunit alpha	-2,66	down
hp1016	*pgsA*	phosphatidylglycerophosphate synthase	-2,66	down
hp0371	*accB*	acetyl-CoA carboxylase biotin carboxyl carrier protein	-2,25	down
hp1348	*plsC*	1-acyl-glycerol-3-phosphate acyltransferase	-2,24	down
hp0559	*acpP*	acyl carrier protein	-2,23	down
hp0700	*dgkA*	diacylglycerol kinase	-2,20	down
hp0561	*fabG*	3-ketoacyl-acyl carrier protein reductase	-2,11	down
hp0871	*cdh*	CDP-diacylglycerol diphosphatase	5,77	up
hp0090	*fabD*	ACP S-malonyltransferase	2,68	up
hp0690	*fadA*	acetyl-CoA acetyltransferase (thiolase)	2,25	up
Transporters				
hp1174	*gluP*	glucose/galactose transporter	4,11	up
hp0715	*lptB*	LPS export ABC transporter ATP-binding protein	3,38	up
hp0133	*sdaC*	HAAAP family serine/threonine permease	2,92	up
hp0251	*oppC*	oligopeptide ABC transporter permease	2,89	up
hp0759		MATE family efflux transporter	2,55	up
hp0613		ABC transporter ATP-binding protein	2,51	up
hp1180	*nupC*	bacterial concentrative nucleoside transporter	2,40	up
hp0942	*dagA*	D-alanine glycine permease	2,35	up
hp1168	*cstA*	carbon starvation protein A	2,31	up
hp1290	*pnuC*	nicotinamide riboside transporter PnuC	2,30	up
hp0140	*lctP*	L-lactate permease	2,28	up
hp0983		small conductance mechanosensitive channel	2,25	up
hp0693		short-chain fatty acids transporter	2,23	up
hp1400	*fecA*	Fe^{3+} dicitrate transport protein (fecA)	2,15	up
hp0818	*proWX*	Osmoprotectant ABC transporter permease	2,14	up
hp0724	*dcuA*	anaerobic C4-dicarboxylate transporter	2,13	up
hp0686	*fecA*	Fe^{3+} dicitrate transport protein	2,03	up
hp0490	*hpkchA*	voltage-gated potassium channel	-3,42	down
hp0582	*tonB1*	Periplasmic protein TonB	-2,67	down
hp1498		LPS export system permease protein	-2,54	down
hp0687	*feoB*	ferrous iron transport protein B	-2,48	down
hp0229	*hopA*	outer membrane porin HopA	-2,40	down
hp1486		ABC transporter permease	-2,19	down

hp1341	*tonB2*	Periplasmic protein TonB	-2,15	down
Virulence				
hp0439		Bacterial virulence protein VirB8 domain-containing protein	4,92	up
hp0310	*pgdA*	peptidoglycan-N-acetylglucosamine deacetylase	4,68	up
hp0547	*cagA*	Type IV secretion system oncogenic effector CagA	3,78	up
hp0017	*virB4a*	VirB4 family type IV secretion/conjugal transfer ATPase	3,68	up
hp0751	*flaG*	polar flagellin FlaG	3,16	up
hp0752	*fliD*	flagellar hook-associated protein 2	2,30	up
hp0520	*cag1*	cag pathogenicity island protein Cag1	2,97	up
hp0019	*cheV1*	chemotaxis protein CheV1	2,88	up
hp1238	*amiF*	formamidase	2,65	up
hp1243	*babA*	Hop family adhesin BabA	2,55	up
hp0541	*cag20*	cag pathogenicity island protein Cag20	2,40	up
hp0243	*napA*	neutrophil-activating protein NapA	2,35	up
hp0896	*babB*	Hop family adhesin BabB	2,24	up
hp0601	*flaA*	flagellin A	2,16	up
hp1399	*rocf*	arginase	2,12	up
hp1086	*tlya*	hemolysin	2,07	up
hp0542	*cag21*	cag pathogenicity island protein Cag21	2,05	up
hp1186		carbonic anhydrase	2,04	up
hp1052	*envA*	UDP-3-O-acyl-N-acetylglucosamine deacetylase	2,02	up
hp0069	*ureF*	urease accessory protein UreF	-4,03	down
hp0725	*sabA*	Hop family adhesin SabA	-3,26	down
hp1203a	*secE*	preprotein translocase subunit SecE	-3,12	down
hp0546a	*picA*	cag pathogenicity island protein	-3,01	down
hp0068	*ureG*	urease accessory protein UreG	-2,89	down
hp1039		O-antigen ligase	-2,76	down
hp0492		neuraminyllactose-binding hemagglutinin	-2,56	down
hp0855	*algl*	alginate O-acetyltransferase complex protein A	-2,55	down
hp0327	*pseH*	UDP-4-amino-4,6-dideoxy-N-acetyl-beta-L-altrosamine N-acetyltransferase	-2,53	down
hp0544	*cag23*	cag pathogenicity island type IV secretion system ATPase CagE	-2,51	down
hp1578		α-(1,2)-N-acetylglucosyltransferase	-2,48	down
hp0280	*lpxL*	lipid A biosynthesis lauroyl acyltransferase	-2,29	down
hp1274	*pflA*	paralysed flagella protein	-2,27	down
hp0508	*pgbA*	plasminogen-binding protein PgbA	-2,24	down
hp1034	*ylxH*	flagellar biosynthesis protein FlhG	-2,20	down
hp0526	*cag6*	cag pathogenicity island translocation protein CagZ	-2,18	down
hp0244	*flgS*	acid survival sensor histidine kinase	-2,18	down
hp0190	*clsC*	Cardiolipin synthase	-2,16	down
hp0410	*hpaA2*	neuraminyllactose-binding hemagglutinin	-2,10	down
hp0256	*fliJ*	flagellar export protein FliJ	-2,10	down

hp0523	*cag4*	cag pathogenicity island protein (cag4)	-2,10	down
hp1006	*virD4*	Type IV secretion system protein VirD4	-2,10	down
hp1462		flagellar motility protein	-2,04	down
Chaperones and heat shock proteins				
hp0109	*dnaK*	molecular chaperone DnaK	3,26	up
hp0264	*clpB*	ATP-dependent Clp protease ATP-binding subunit ClpB	3,23	up
hp0110	*grpE*	nucleotide exchange factor GrpE	3,22	up
hp0010	*groEL*	chaperonin GroEL	2,74	up
hp1024	*cbpA*	co-chaperone-curved DNA binding protein A	2,67	up
hp0210	*htpG*	molecular chaperone HtpG	2,04	up
tRNA biosynthesis				
hpt28	*tRNA-Met-1*	Transfer RNA	4,41	up
hpt09	*tRNA-Ala-1*	Transfer RNA	3,71	up
hpt20	*tRNA-Met-3*	Transfer RNA	3,67	up
hpt34	*tRNA-Leu-2*	Transfer RNA	3,53	up
hpt35	*tRNA-Leu-3*	Transfer RNA	3,15	up
hpt17	*tRNA-Arg-3*	Transfer RNA	2,86	up
hpt02	*tRNA-Asp-1*	Transfer RNA	2,74	up
hpt10	*tRNA-Arg-1*	Transfer RNA	2,72	up
hpt01	*tRNA-Glu-1*	Transfer RNA	2,71	up
hpt08	*tRNA-Asn-1*	Transfer RNA	2,67	up
hpt26	*tRNA-Pro-1*	Transfer RNA	2,67	up
hpt27	*tRNA-Ser-2*	Transfer RNA	2,58	up
hpt21	*tRNA-Gln-1*	Transfer RNA	2,51	up
hpt31	*tRNA-Gly-2*	Transfer RNA	2,40	up
hpt11	*tRNA-Ala-2*	Transfer RNA	2,23	up
hpt18	*tRNA-Met-2*	Transfer RNA	2,16	up
hpt15	*tRNA-His-1*	Transfer RNA	2,12	up
hp0774	*tyrS*	tyrosyl-tRNA synthetase	2,06	up
hp0830	*gatA*	Glu-tRNA(Gln) amidotransferase, subunit A	2,04	up
hp1241	*alaS*	alanyl-tRNA synthetase	2,00	up
hp1448	*rnpA*	ribonuclease P protein component	-2,92	down
hp1148	*trmD*	tRNA (guanine-N1)-methyltransferase	-2,51	down
hp1452	*mnmE*	tRNA modification GTPase	-2,28	down
hp0182	*lysS*	lysyl-tRNA synthetase	-2,08	down
Vitamins, secondary metabolites, and cofactors				
hp1291		thiamine diphosphokinase	4,40	up
hp0006	*panC*	pantoate--beta-alanine ligase	2,53	up
hp0845	*thiM*	hydroxyethylthiazole kinase	2,45	up
hp0844	*thi*	bifunctional hydroxymethylpyrimidine kinase/phosphomethylpyrimidine kinase	2,29	up

hp0034	*panD*	aspartate 1-decarboxylase	2,05	up
hp0761		5-formyltetrahydrofolate cyclo-ligase	2,01	up
hp1545	*folC*	bifunctional folylpolyglutamate synthase/dihydrofolate synthase	-2,90	down
hp1224	*hemD*	Uroporphyrinogen-III synthase	-2,23	down
hp0934	*queE*	7-carboxy-7-deazaguanine synthase	-2,13	down
hp0769	*mobA*	molybdenum cofactor guanylyltransferase MobA	-2,04	down
Metal resistance				
hp1073	*copP*	copper-binding metallochaperone CopP	-2,57	down
hp1488		efflux RND transporter periplasmic adaptor subunit	-2,27	down
hp1072	*copA*	Cu^{2+} exporting P-type ATPase CopA	-2,14	down
hp0971	*cznC*	Ni^{2+}/Zn^{2+}/Cd^{2+} efflux pump, outer membrane channel	-2,13	down
hp0653	*pfr*	ferritin	4,73	up
hp1326	*crdA*	copper resistance determinant CrdA	2,05	up
Electron transfer and oxidative phosphorylation				
hp0377	*resA*	dithiol reductase/isomerase ResA	-2,58	down
hp1137	*atpX*	F0F1 ATP synthase, subunit B′	-2,43	down
hp0378	*ccsbA*	bifunctional cytochrome c biogenesis protein CcsBA	-2,26	down
hp1135	*atpH*	F0F1 ATP synthase, subunit D	-2,23	down
hp0828	*atpB*	F0F1 ATP synthase, subunit A	-2,12	down
hp1227	*cyt553*	cytochrome c-553	-2,07	down
hp1273	*nuoN*	NADH-quinone oxidoreductase subunit NuoN	-2,04	down
hp1136	*atpF*	F0F1 ATP synthase, subunit B	-2,03	down
hp0193	*frdC*	fumarate reductase, cytochrome b subunit	-2,01	down
hp0631	*hydA*	Quinone-reactive Ni/Fe hydrogenase	4,06	up
hp0632	*hydB*	Quinone-reactive Ni/Fe hydrogenase	3,66	up
hp0633	*hydC*	Ni/Fe-hydrogenase, b-type cytochrome subunit	3,08	up
Restriction and modification systems				
hp1402	*hsdR3*	Type I restriction endonuclease subunit R	4,51	up
hp0262		Type II restriction enzyme	2,90	up
hp1208		adenine-specific DNA-methyltransferase	2,30	up
hp0091	*hsdR*	Type II restriction endonuclease	2,13	up
hp1517		DNA methyltransferase	2,09	up
hp1403	*hsdM*	Type I restriction enzyme M protein	2,05	up
hp0592	*hpyAXIR*	Type III restriction-modification system endonuclease	-3,44	down
hp0050	*dpnA*	adenine specific DNA-methyltransferase	-2,05	down
Replication, repair, recombination				
hp1460	*dnaE*	DNA polymerase III subunit alpha	3,18	up
hp1470	*polA*	DNA polymerase I	2,48	up
hp0438	*tnpB*	transposase	2,14	up
hp1114	*uvrB*	excinuclease ABC subunit B (UvrB)	2,04	up

hp0827		Single-strand DNA-binding protein (SSB)	-5,38	down
hp1231	*holB*	DNA polymerase III subunit delta	-2,96	down
hp1347	*ung*	uracil-DNA glycosylase	-2,30	down
Translation factors				
hp0077	*prfA*	peptide chain release factor 1	-6,12	down
hp1298	*infA*	translation initiation factor IF-1	-4,62	down
hp1441	*ppiA*	peptidyl-prolyl cis-trans isomerase B	-3,06	down
hp0795	*tig*	trigger factor	-2,49	down
hp1497	*pth*	peptidyl-tRNA hydrolase	-2,21	down
hp1123	*slyD*	peptidyl-prolyl cis-trans isomerase	-2,09	down
hp0124	*infC*	translation initiation factor IF-3	-2,02	down
Transcription factors				
hp1025	*hspR*	MerR family transcriptional regulator HspR	3,79	up
hp0111	*hrcA*	HrcA family transcriptional regulator	3,47	up
hp0714	*rpoN*	RNA polymerase factor sigma-54	2,06	up
hp0866	*greA*	transcription elongation factor GreA	2,01	up
hp1203	*nusG*	transcription termination factor NusG	-2,36	down
hp0550	*rho*	transcription termination factor Rho	-2,26	down
Outer membrane proteins				
hp1342	*hopN*	Hop family outer membrane protein HopN	3,16	up
hp0227	*hopM*	Hop family outer membrane protein HopM	3,09	up
hp0317	*omp9*	outer membrane beta-barrel protein	2,73	up
hp0472	*omp11*	outer membrane beta-barrel protein	2,46	up
hp1395	*horL*	outer membrane protein HorL/Omp30	2,22	up
hp1456	*lpp20*	LPP20 family lipoprotein	2,16	up
hp1066		Outer membrane protein	2,08	up
Antioxidant enzymes				
hp0875	*katA*	catalase	3,11	up
hp0389	*sodB*	superoxide dismutase [Fe]	2,43	up
hp1461	*ccP*	cytochrome c551 peroxidase	2,32	up
hp0407	*bisC*	Biotin sulfoxide reductase	2,10	up
Nucleotide metabolism				
hp1218a	*purD*	glycinamide ribonucleotide synthetase	5,94	up
hp1240		nucleoside triphosphate pyrophosphatase	3,11	up
hp1178	*deoD*	purine-nucleoside phosphorylase	2,38	up
hp0735	*gpt*	xanthine guanine phosphoribosyl transferase	-2,28	down
Peptidases				
hp1069	*ftsH*	ATP-dependent zinc metalloprotease FtsH	3,77	up
hp0570	*pepA*	leucyl aminopeptidase	3,65	up

hp1299	*map*	methionine amino peptidase	-2,04	down
**Others**				
hp1450	*yidC*	YidC/Oxa1 family membrane protein insertase	-3,72	down
hp0645	*slt*	peptidoglycan lytic transglycosylase	-3,36	down
hp0331	*minD*	septum site-determining protein MinD	2,15	up
hp1198	*rpoB*	DNA-directed RNA polymerase, beta subunit	2,01	up

[1]Gene annotations and functional categories were assigned according to the information stored in the databases KEEG (https://www.genome.jp/kegg/), BioCyc (https://biocyc.org/), and UniProt (https://www.uniprot.org/).

Table S4. List of oligonucleotides used in this study.

Oligo ID	Sequence 5´- 3´	Use
HsrA_up	GGAATTCCATATGCGCGTTCTACTGATTG	Cloning *hsrA*
HsrA_dw	CCCAAGCTTTTACTCTTCACACGCCGG	Cloning *hsrA*
CosR_up	GGAATTCCATATGAGAATTTTAGTTATAGAAG	Cloning *cosR*
CosR_dw	CGGGATCCTTAAGATTTTTAGGGAAGCAGAAACGG	Cloning *cosR*
PporG_up	CCCCACACTTGCCCCATACAGAC	EMSA
PporG_dw	GCATGCCATCTAATTTGAAACATGG	EMSA
PsodB_up	CTGCGAAAGCACCTAGTAATGC	EMSA
PsodB_dw	GTAACATAAGTATTGTGATGTTTTCCATG	EMSA
pkn22_up	CGGTCAAGCTGTACAGCAGTCAG	EMSA
pkn22_dw	GAACGCCATAGCCTACAGTAGGC	EMSA
qPCR-glnAup	CGGATGTGAGCGTGGTCGTG	qPCR
qPCR-glnAdw	GCCACATCGCCCAAACCTGAATC	qPCR
qPCR-rpmIup	CGGCGCGTCTAAGCGTTTCAAAG	qPCR
qPCR-rpmIdw	CGCGTTTAGATTGGCTTTGCGC	qPCR
qPCR-tlpBup	GAGCATGAAAGATTCCTCAACCACC	qPCR
qPCR-tlpBdw	CGTTTCAATCAAACGCTTCCTTAACC	qPCR
qPCR-rplUup	GGAAGTGTTAGCCGTATCCAAAGAGGG	qPCR
qPCR-rplUdw	GCCGCGCCCTTCATTGATCAC	qPCR
qPCR-rpmAup	GATTCTGCAGGAAGACGCTTAGGCG	qPCR
qPCR-rpmAdw	CCCATGCCCACATTATTACCAGGATGC	qPCR
qPCR-sodBup	GCGACTGCCCTAAGCGATGAG	qPCR
qPCR-sodBdw	GTGGTCGCGCTCTTAATGAAGTC	qPCR
qPCR-16SRNAup	CTGAGAGGGTGAACGGACACACTG	qPCR
qPCR-16SRNAdw	CGTTGCTGCTTCAGGGTTTCCC	qPCR
qPCR-dnaEup	CAGCGCCGGCGTAAGGAAATC	qPCR
qPCR-dnaEdw	CTTGCGACATCTCTGATCACGCC	qPCR
qPCR-cagAup	CGGCAGTGGCTTAGTCATAGCAGG	qPCR
qPCR-cagAdw	GACTCAATGCTCGTTGTGAGCCTG	qPCR
qPCR-pfr-up	GCTTAGATGGCGCGGGGC	qPCR
qPCR-pfr-dw	GATGCTAGTCAATTGCACAGGCACATTG	qPCR
qPCR-cdh-up	GACTATGGCTTAGCGGTGGTGC	qPCR
qPCR-cdh-dw	GAATCTCTTCGGCTGAAGCGCG	qPCR
